# *Centella asiatica* mitigates the detrimental effects of Bisphenol-A (BPA) on pancreatic islets

**DOI:** 10.1038/s41598-024-58545-2

**Published:** 2024-04-05

**Authors:** Oly Banerjee, Siddhartha Singh, Tiyesh Paul, Bithin Kumar Maji, Sandip Mukherjee

**Affiliations:** 1 Department of Physiology, Serampore College, 9 William Carey Road, Serampore, Hooghly, West Bengal 712201 India; 2 Department of Medical Laboratory Technology, School of Allied Health Sciences, Swami Vivekananda University, Bara Kanthalia, West Bengal 700121 India

**Keywords:** Bisphenol A (BPA), Insulin resistance, *Centella asiatica*, Oxidative stress, Inflammation, Apoptosis, Biochemistry, Molecular biology, Natural hazards, Endocrinology

## Abstract

Bisphenol-A (BPA) is widely used in food packaging and household products, leading to daily human exposure and potential health risks including metabolic diseases like type 2 diabetes mellitus (T2DM). Understanding BPA's mechanisms and developing intervention strategies is urgent. *Centella asiatica*, a traditional herbal medicine containing pentacyclic triterpenoids, shows promise due to its antioxidant and anti-inflammatory properties, utilized for centuries in Ayurvedic therapy. We investigated the effect of *Centella asiatica* (CA) ethanol extract on BPA-induced pancreatic islet toxicity in male Swiss albino mice. BPA administration (10 and 100 μg/kg body weight, twice daily) for 21 days caused glucose homeostasis disturbances, insulin resistance, and islet dysfunction, which were partially mitigated by CA supplementation (200 and 400 mg/kg body weight). Additionally, heightened oxidative stress, elevated levels of proinflammatory cytokines, loss of mitochondrial membrane potential (MMP), abnormal cell cycle, and increased apoptosis were implicated in the detrimental impact of BPA on the endocrine pancreas which were effectively counteracted by CA supplementation. In summary, CA demonstrated a significant ability to mitigate BPA-induced apoptosis, modulate redox homeostasis, alleviate inflammation, preserve MMP, and regulate the cell cycle. As a result, CA emerged as a potent agent in neutralizing the diabetogenic effects of BPA to a considerable extent.

## Introduction

Diabetes stands as a formidable global public health challenge, casting a substantial burden on both public health and socio-economic development worldwide. Ranked among the top 10 causes of mortality on a global scale, diabetes is a critical concern. According to estimates from the International Diabetes Federation (IDF), the global diabetic population numbered 536.6 million adults in 2021, with projections soaring to 783.2 million by 2045 if effective preventive measures are not implemented^1^. The presence of diabetes is linked to heightened mortality rates associated with infections, cardiovascular disease, stroke, chronic kidney disease, chronic liver disease, and cancer^2,3^. Despite strides in advancing population health and prolonging life expectancy, diabetes remains the second most significant factor negatively impacting global health-adjusted life expectancy^4^.

Numerous countries worldwide have implemented strategies to address behavioral risk factors, focusing on promoting healthy lifestyles, smoking cessation, maintaining a low-fat diet, combating the fast-food culture, and encouraging physical activity to mitigate the high prevalence of diabetes. However, these intervention efforts often overlook the role of occupational-related environmental pollution^5^. Additionally, environmental toxicants, functioning as endocrine-disrupting chemicals (EDCs), have emerged as novel risk factors for metabolic diseases^6^. Among these substances, bisphenol-A (BPA), a key component in the production of polycarbonate plastic and epoxy resins, has been identified as a potential link between chemical exposure and the development of type 2 diabetes mellitus (T2DM)^7^. Bisphenol-A (BPA) has widespread use in food packaging, disposable water bottles, and coatings for tin cans, food containers, drinking glasses, bowls, cups, and microwave-safe utensils resulting in day-to-day human exposure. General population can also be exposed through dust particle inhalation^8^. The widespread prevalence of BPA exposure is also evident in its high detection frequency in human blood and urine samples^9^. Evidence from human, terrestrial, and aquatic models has consistently linked environmentally or human-relevant doses of BPA exposure to adverse metabolic consequences, including T2DM^10^. Experimental animal study have affirmed that environmentally relevant doses of BPA exposure can lead to biological effects such as islet cell dysfunction and insulin resistance (IR) in mice^11^. Various factors contribute to the development of IR, including an increase in elements that negatively impact insulin sensitivity, such as chronic low-grade inflammation, dyslipidemia, oxidative stress, and lipotoxicity in metabolic tissues^12^. Similarly, an impaired oxidant–antioxidant balance and an elevated inflammatory response are proposed as potential mechanisms for BPA's diabetogenic effects^13^.

Although robust evidence underscores the detrimental impact of BPA on pancreatic islets and glucose homeostasis, clear guidance on minimizing these effects remains elusive. The widespread presence of endocrine disruptors in all stages of food cultivation, transportation, storage, and preparation results in exposure across the living world, encompassing humans, terrestrial, and aquatic animals. BPA carries the potential for life-altering consequences on biological systems, particularly in humans. Consequently, prompt global action is imperative to mitigate systematic exposure in all aspects of modern living.

*Centella asiatica* (L.) Urb. is a perennial herbaceous creeper belonging to the Apiaceae family. It is rich in triterpenes, including asiatic acid, madecassic acids, asiaticoside, and madecassoside^14^, as well as essential oils, amino acids, and various other compounds. *Centella asiatica* is indigenous to India, China, Sri Lanka and Australia as well as South Africa (including African islands Madagascar and Seychelles) and South pacific and Eastern Europe^15^. This herb has been employed in traditional medicine across diverse communities worldwide, with reported uses spanning dermal problems, neurodegenerative diseases, digestive disorders, metabolic disorders, among others^16^. Moreover, *Centella asiatica* has historically been utilized in traditional medicine across Asian and African nations for addressing various health concerns, such as diabetes^15^. Today, it is readily accessible as a dietary supplement on the market, for example, powdered *Centella asiatica* extract [U.S. Pharmacopeia (USP)], Glotu Cola-Solger: PhytO2X^®^ [Iswari Superfood, Portugal], Organic Gotu Kola Tablets (*Centella Asiatica*) [Just Jaibik. India] etc. Notably, *Centella asiatica* exhibits robust antioxidant and anti-inflammatory properties^17^. *Centella asiatica* contains a high level of antioxidant from the group of pentacyclic triterpenoids (asiatic acid, madecassic acid, asiaticoside, and madecassoside)^18,19^. The content of triterpenoid contained in *Centella asiatica* is known to improve glucose response through increased protein GLUT4, IR, IRS 1, and IRS 2^20,21^. The presence of inflammatory response, oxidative stress, apoptosis, and mitochondrial dysfunction is intricately linked to islet cell dysfunction and insulin resistance across various conditions with diverse origins^22^. *Centella asiatica* and its triterpenoids exhibit utility in numerous pathological scenarios due to their anti-inflammatory and anti-apoptotic effects, alleviation of oxidant stress, and enhancement of mitochondrial function. Given its intriguing biological attributes, further exploration of this medicinal herb is warranted, particularly regarding its potential impact on the diabetogenic action of BPA.

In the interim, the objective of the current study is to examine the potential beneficial effects of *Centella asiatica* on countering the toxic impact of BPA on pancreatic islets and impaired glucose homeostasis. It is anticipated that the findings from this study will serve as a valuable resource for individuals, healthcare providers, and governmental bodies. This research aims to empower them to make informed, evidence-based decisions aimed at mitigating the adverse effects of BPA on the well-being of their populations.

## Results

### Triterpene contents in ethanol and methanol extract of *Centella asiatica*

According to the HPLC analysis, the ethanol extract of *Centella asiatica* at a concentration of 5 mg/ml exhibits a higher quantity of triterpenes compared to the methanol extract (Figure S1). Among the three distinct triterpenes analyzed, asiatic acid is the most abundant in both the ethanol (48.0 µg/ml) and methanol (38.0 µg/ml) extracts, followed by asiaticoside (47.0 µg/ml in ethanol extract and 24.0 µg/ml in methanol extract) and madecassic acid (33.0 µg/ml in ethanol extract and 25.0 µg/ml in methanol extract). Due to its superior triterpene content, we opted for the ethanol extract of *Centella asiatica* (CA) for supplementation in BPA-exposed animals because triterpenes are the main chemical components responsible for its pharmacological activity, especially asiaticoside, asiatic acid, madecassoside, and madecassic acid^23^.

### Acute toxicity study

In the acute toxicity study, male mice received the ethanol extract of *Centella asiatica* (CA) via oral gavage. They were observed for any alterations in appearance and behavior, including restlessness, drowsiness, piloerection, writhing, convulsions, and mortality. Monitoring occurred within the first 4 h, intermittently over the subsequent 24 h, and then daily for a total of 14 days. The outcomes of the acute toxicity study affirm the safety of *Centella asiatica* (CA) for oral consumption, with no adverse effects observed at doses up to 4000 mg/kg of body weight. Importantly, no instances of mortality or observable toxic reactions (changes in behavior, fur condition, nails colour, eye colour, convulsion, locomotion, dysponea, sedation, and aggression) were noted during this comprehensive monitoring period (Table S1).

### BPA mediated changes in glucose homeostasis: role of CA

**Figure 1:**
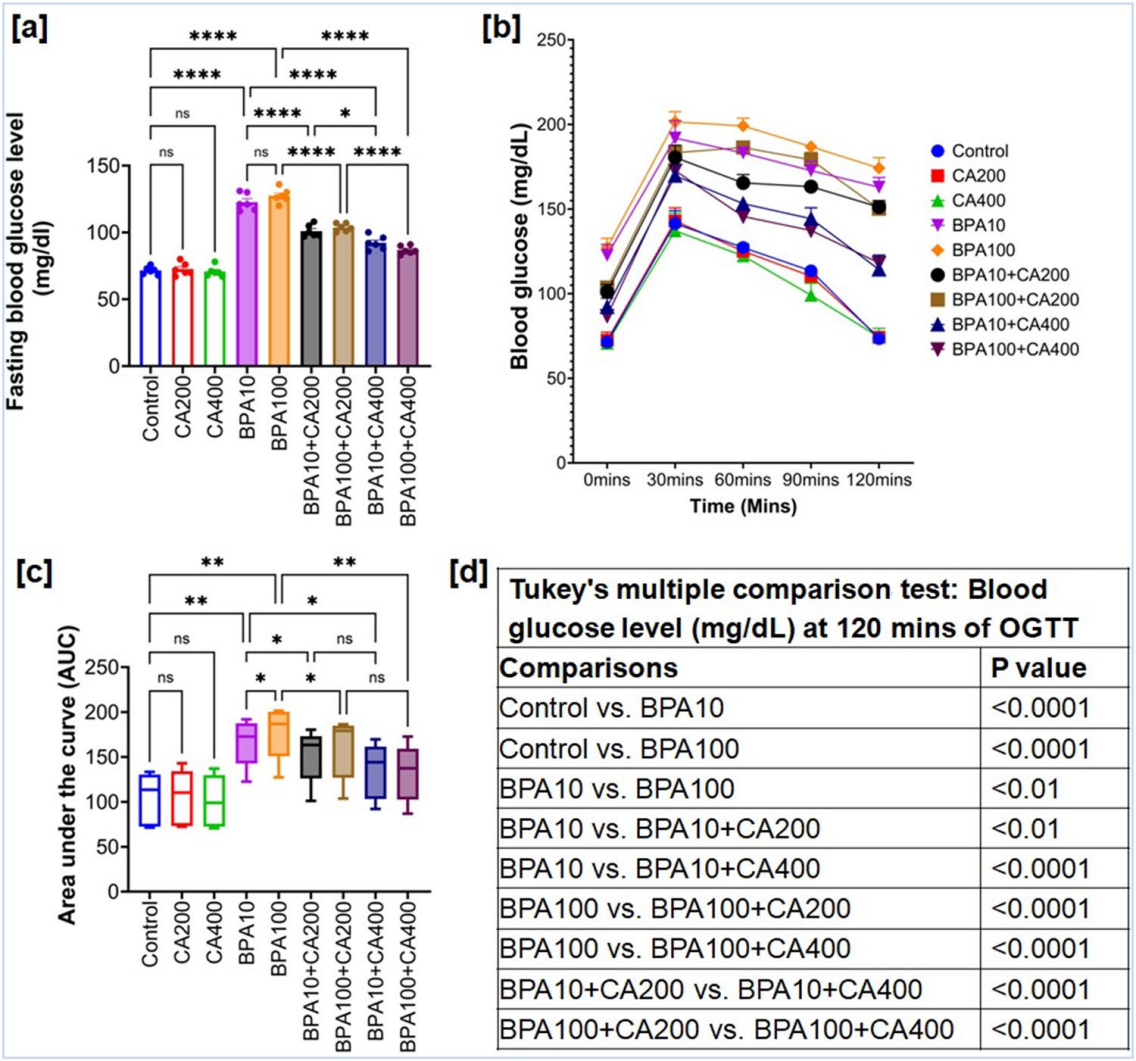
Protective efficacy of CA against BPA-mediated impaired fasting blood glucose (FBG) level and oral glucose tolerance test (OGTT). (**a**) FBG, (**b**) OGTT and (**c**) area under the curve (AUC) for OGTT in BPA (10 and 100 µg/kg body weight for 21 days) treated mice with or without supplementation of CA (200 and 400 mg/kg body weight/day for 21 days). Data were presented as mean ± SEM (n = 6). Normality of data was tested by Shapiro–Wilk test. Significance level based on one-way ANOVA (for FBG and AUC), p < 0.05, or two-way ANOVA (for OGTT), p < 0.05. Significance level based on Tukey’s post hoc test *p < 0.05, ** p < 0.01, ***p < 0.001, ****p < 0.0001, ns—not significant. (**d**) Significance level based on Tukey’s multiple comparison test for blood glucose level during OGTT at 120 min.

Impairments in insulin production or uptake can amplify the presence of impaired blood glucose levels, and elevated fasting blood glucose (FBG) levels indicate a vulnerable state of pancreatic β cells in insulin-resistant conditions^24^. This has motivated us to assess FBG levels in animals treated with BPA, both with and without CA supplementation. In Fig. 1a, it is evident that FBG levels in BPA-exposed mice were significantly elevated (p < 0.0001) compared to control mice after 21 days of exposure. Notably, supplementation with CA resulted in a marked reduction in FBG levels. Furthermore, treatment of CA at a dose of 400 mg/kg demonstrated a more pronounced decrease in FBG levels in BPA-exposed mice compared to control mice (p < 0.0001).

Further, in order to assess the potential for BPA exposure to cause damage to islets and disrupt blood glucose regulation, an oral glucose tolerance test (OGTT) was conducted. In Fig. 1b,c, the results of the OGTT and area under curve (AUC) for oral glucose tolerance in BPA-exposed mice are presented. It was observed that BPA-exposed mice reached a peak in blood glucose levels after 30 min following the oral glucose challenge, and these elevated levels persisted up to 120 min, in contrast to the control group mice (p < 0.0001). However, when CA was supplemented to BPA-treated mice, the peak blood glucose level occurred at 30 min after the glucose load, resembling the values expected in the control group, and by 120 min, it had approached a reasonable alignment with the control values (Fig. 1b,d). Likewise, AUC for oral glucose tolerance was also significantly increased in BPA-treated mice (p < 0.01) and CA supplementation exerts significant protection against these BPA-induced changes (low dose: p < 0.05, high dose: p < 0.01).

**Figure 2:**
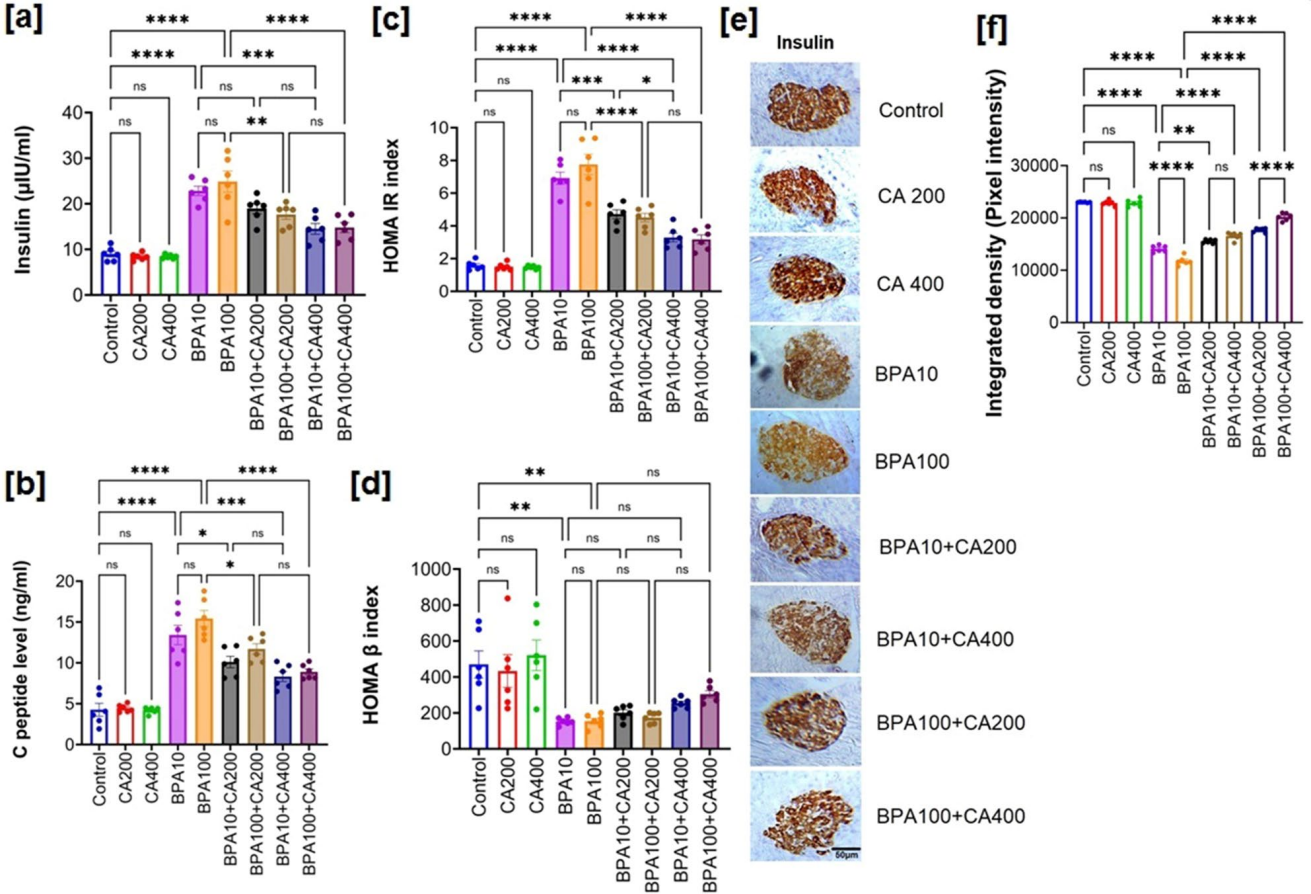
CA supplementation extends protection against BPA-induced pancreatic islet cell dysfunction and insulin resistance. Changes in plasma insulin and c-peptide level, HOMA-IR and HOMA-β index and insulin expression in pancreatic islets on exposure to BPA (10 and 100 µg/kg body weight for 21 days) with or without supplementation of CA (200 and 400 mg/kg body weight/day for 21 days): (**a**) fasting plasma insulin level, (**b**) fasting C-peptide level, (**c**) HOMA-IR index and (**d**) HOMA-β index. Data were presented as mean ± SEM (n = 6). Normality of data was tested by Shapiro–Wilk test. Significance level based on one-way ANOVA, p < 0.05. Significance level based on Tukey’s post hoc test *p < 0.05, **p < 0.01, ***p < 0.001, ****p < 0.0001, *ns* not significant. (**e**) Representative photomicrograph of immunohistochemical analysis of insulin in pancreatic islets of BPA (10 and 100 µg/kg body weight for 21 days) treated mice with or without supplementation of CA (200 and 400 mg/kg body weight/day for 21 days). Results are representative of six mice. Magnification ×200 and scale bar: 50 µm for all panels. (**f**) Quantification of immunohistochemical images presented in (**e**) by Image J software and expressed as integrated density (pixel density). Data were presented as mean ± SEM (n = 6). Normality of data was tested by Shapiro–Wilk test. Significance level based on one-way ANOVA, p < 0.05. Significance level based on Tukey’s post hoc test *p < 0.05, **p < 0.01, ***p < 0.001, ****p < 0.0001, *ns* not significant.

Figure 2a,b illustrate notable findings regarding plasma insulin and c-peptide levels in BPA-treated mice, revealing a significant increase compared to control mice (p < 0.0001). The supplementation of CA at two different dosages (200 and 400 mg/kg body weight per day) demonstrated the capacity to bring down these elevated plasma insulin and c-peptide levels, approaching values similar to those of the control group. Notably, the 400 mg/kg dose of CA exhibited significant efficacy (p < 0.0001).

As insulin resistance is closely associated with increased fasting insulin and c-peptide levels during the early stages of diabetes, we computed the homeostasis model assessment of insulin resistance (HOMA IR) and homeostasis model assessment of beta cell function (HOMA-β) indices in BPA-exposed mice (Fig. 2c,d). The HOMA-IR and HOMA-β, which are based on plasma levels of fasting glucose and insulin, have been extensively validated and utilized for measuring insulin resistance and β-cell function^25^. Elevated HOMA-IR and diminished HOMA-β have been consistently linked, independently, to an elevated risk of diabetes^25^. Furthermore, reduced HOMA-β has been consistently associated with an increased diabetes risk, irrespective of HOMA-IR levels^26^. The administration of BPA for 21 days resulted in a significantly higher HOMA-IR score (p < 0.0001), while the HOMA-β index was markedly lower (p < 0.01) compared to the control mice. Importantly, the supplementation of CA at two graded doses demonstrated an improvement in the HOMA-IR score and HOMA-β in BPA-exposed mice, with the 400 mg/kg dose of CA displaying significant efficacy (HOMA IR: p < 0.0001, HOMA β: p < 0.01). Thus, impaired HOMA-IR and HOMA-β indices indicate insulin resistance and β cell dysfunction induced by exposure to BPA.

To corroborate the findings related to insulin and c-peptide levels, we conducted an examination of insulin expression in the pancreatic islets of BPA-exposed mice, both with and without CA supplementation, using immunohistochemistry (Fig. 2e,f). The results of the immunohistochemical staining revealed a noticeable decrease in the expression of insulin in the pancreatic islets of BPA-treated mice compared to those of the control group (Fig. 2e). Significantly, the supplementation of CA, in a dose-dependent manner, had a substantial mitigating effect on the BPA-induced alterations in insulin staining, preserving the normal expression patterns to a considerable extent.

### Effect of CA on histological changes in the pancreatic islets of BPA-treated mice

**Figure 3:**
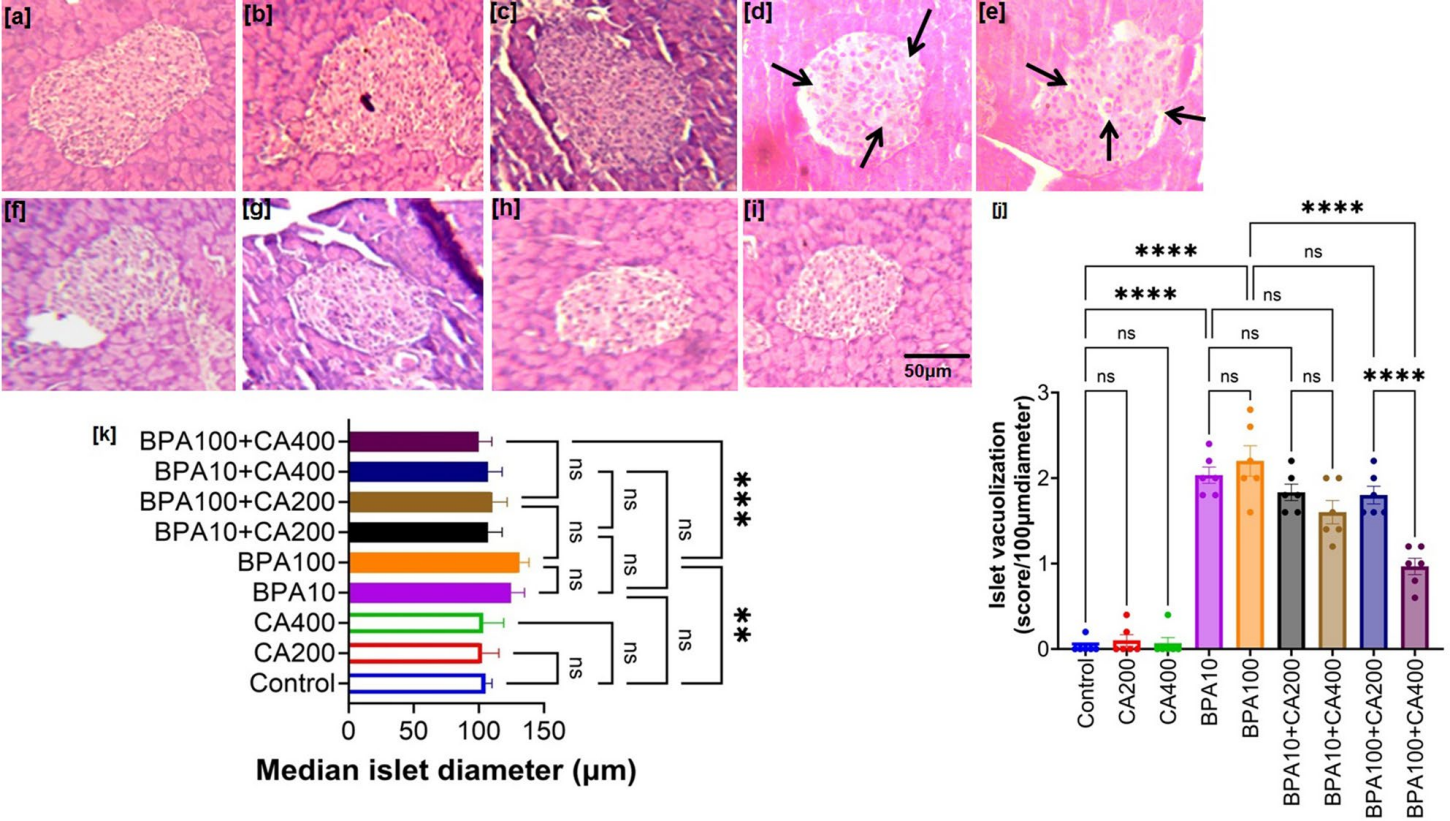
Beneficial impact of CA supplementation on BPA-induced change in pancreatic islet cell morphology. Representative photomicrograph of haematoxylene-eosine stained pancreas sections of BPA (10 and 100 µg/kg body weight for 21 days)-treated mice with or without supplementation of ethanol extract of *Centella asiatica* (CA: 200 and 400 mg/kg body weight/day for 21 days). (**a**) Control, (**b**) CA supplemented (200 mg/kg body weight/day for 21 days), (**c**) CA supplemented (400 mg/kg body weight/day for 21 days), (**d**) BPA treated (10 µg/kg body weight for 21 days), (**e**) BPA treated (100 µg/kg body weight for 21 days), (**f**) BPA10 + CA supplemented (200 mg/kg body weight/day for 21 days), (**g**) BPA10 + CA supplemented (400 mg/kg body weight/day for 21 days), (**h**) BPA100 + CA supplemented (200 mg/kg body weight/day for 21 days) and (**i**) BPA 100 + CA supplemented (400 mg/kg body weight/day for 21 days). Magnification ×400 and scale bar: 50 µm for all panels. (**j**) Quantification of cytoplasmic vacuolization of pancreatic islets. Vacuolization score was calculated using the following: > 2 vacuoles/100 μm islet diameter—1, > 4 vacuoles/100 μm islet diameter—2, > 8 vacuoles/100 μm islet diameter—3 (n = 6; from each animal, 3 islets were considered for scoring). (**k**) Median islet diameter (µm). Data of median islet diameter were presented as mean ± SEM (n = 6). Normality of data was tested by Shapiro–Wilk test. Significance level based on one way ANOVA, P < 0.001. Significance level based on Tukey’s post hoc test *P < 0.05, **P < 0.01, ***P < 0.001, *ns* not significant.

In Fig. 3a–i, pancreas tissue sections from mice exposed to BPA, stained with eosin and hematoxylin, revealed a modified pancreatic structure characterized by increased cytoplasmic vacuolization and diffuse islet cells as compared to control mice. Moreover, quantitative analysis of vacuolization (Fig. 3j) demonstrated that its occurrence increased in both BPA-treated groups, while the supplementation of CA (200 and 400 mg/kg body weight/day) was found to effectively mitigate BPA-mediated cytoplasmic vacuolization of pancreatic islets. Moreover, the median diameter of pancreatic islets (µm) was elevated at both BPA doses, with BPA400 demonstrating significant changes (p < 0.01) (Fig. 3k). The increased median diameter of pancreatic islets observed in mice treated with BPA provides additional evidence of compensatory adaptations within the islets, responding to the loss of islet beta cells, as demonstrated in immunohistochemistry images (Fig. 2e). Thus, we posit that the augmentation in median islet diameter precedes the onset of heightened insulin resistance.

However, the addition of CA at two different doses was found to maintain a typical pancreatic architecture and enhance the number of islet cells in a dose-dependent manner, with the 400 mg/kg dose exhibiting the most notable effectiveness. As anticipated, the median diameter of islet cells was significantly restored by higher concentration of CA supplementation.

### Impact of BPA on lipid profile parameters: role of CA

**Figure 4:**
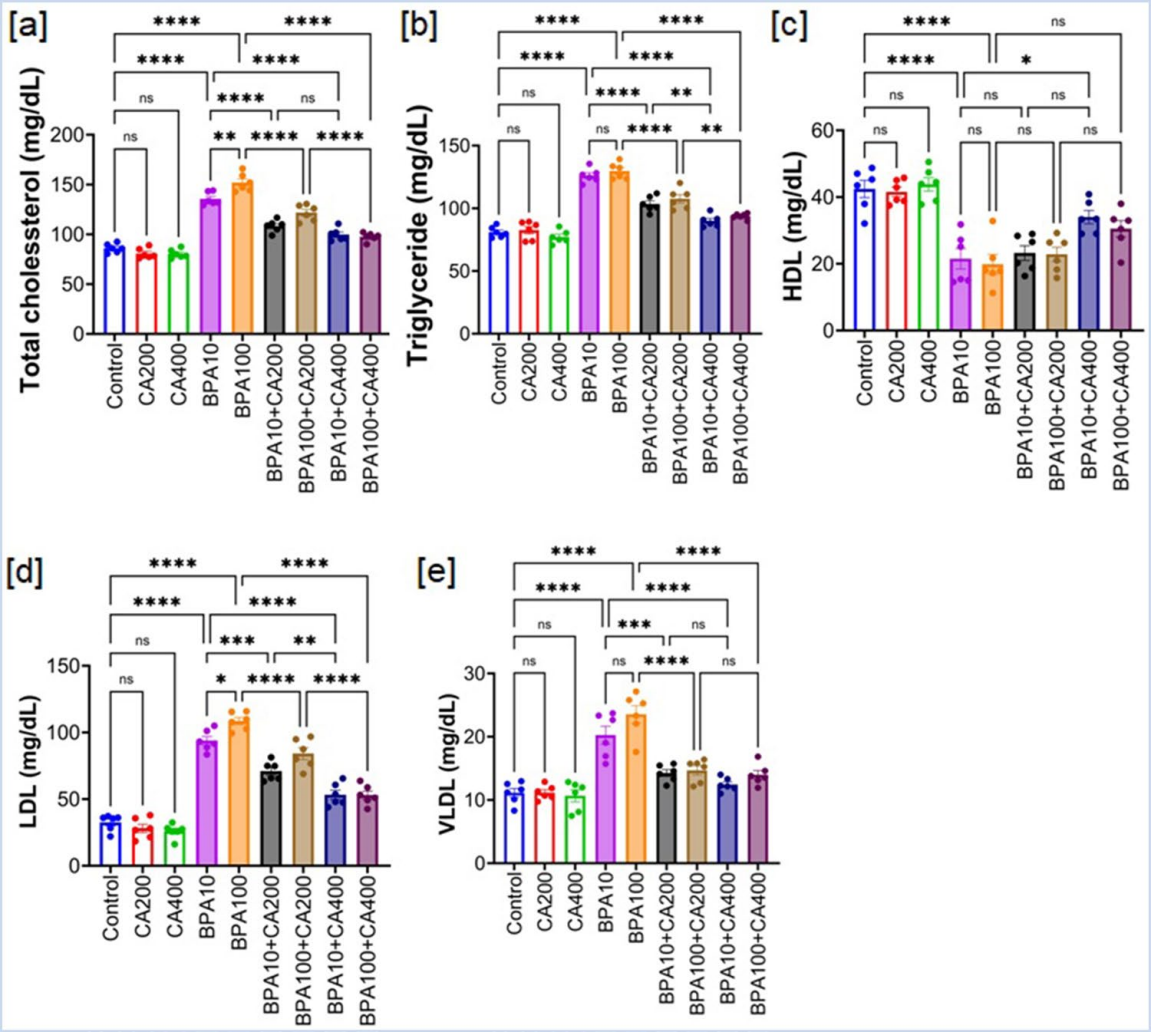
Adverse effect of BPA on lipid profile parameters of mice: protective role of CA. Serum lipid profiles of BPA (10 and 100 µg/kg body weight for 21 days)-treated mice with or without CA supplementation (200 and 400 mg/kg body weight/day for 21 days). (**a**) Total cholesterol (mg/dl), (**b**) triglycerides (mg/dl), (**c**) HDL (mg/dl), (**d**) LDL (mg/dl) and (**e**) VLDL (mg/dl). Data were presented as mean ± SEM (n = 6). Normality of data was tested by Shapiro–Wilk test. Significance level based on one way ANOVA, p < 0.05. Significance level based on Tukey’s post hoc test *p < 0.05, **p < 0.01, ***p < 0.001, ****p < 0.0001, *ns* not significant.

Earlier findings suggest that BPA might contribute to lipid abnormalities, such as dyslipidemia^27,28^. Furthermore, dysregulated lipid metabolism is a key contributor to the development of insulin resistance^29^. To investigate the relationship between BPA-induced lipid abnormalities and insulin resistance, we assessed lipid profile parameters in the serum of BPA-exposed mice with and without supplementation of CA (Fig. 4). The findings revealed that mice exposed to BPA exhibited significantly elevated levels of total cholesterol (TC), triglycerides (TG), low-density lipoprotein (LDL), and very low-density lipoprotein (VLDL), accompanied by a simultaneous reduction in high-density lipoprotein (HDL) levels (p < 0.0001).

Notably, the administration of a higher dose of CA supplementation (400 mg/kg body weight) resulted in a substantial alleviation of BPA-induced dyslipidemia. However, the lower dose of CA (200 mg/kg body weight per day) showed statistically significant improvement in the lipid profile parameters of the BPA-exposed mice except HDL.

### Beneficial effect of CA supplementation on BPA mediated oxidative stress in pancreatic islets

**Figure 5:**
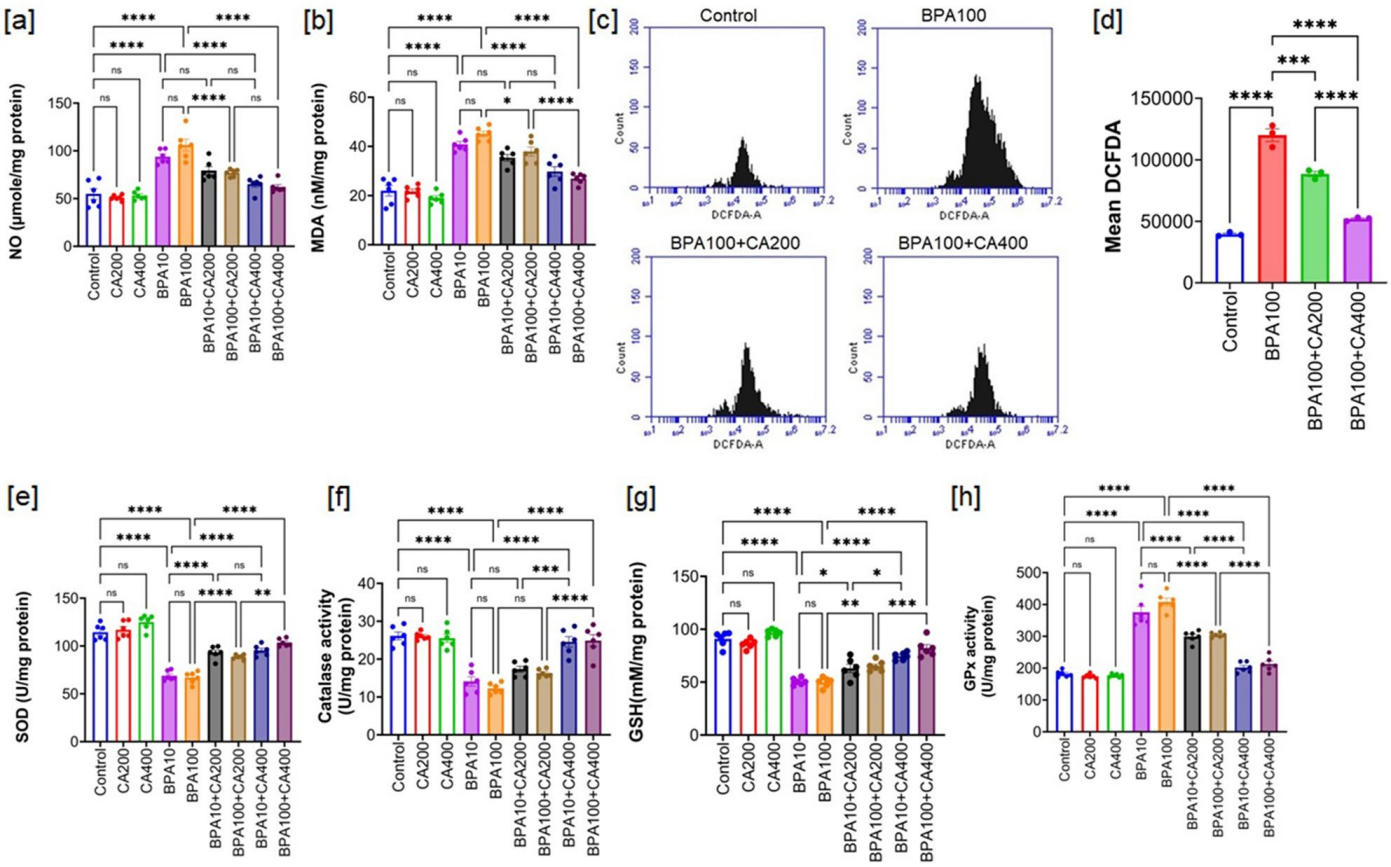
CA regulates oxidative stress in pancreatic islets, enhancing its protective effects against the toxicity induced by BPA. Effect of BPA (10 and 100 µg/kg body weight for 21 days) on oxidative stress parameters with or without supplementation of ethanol extract of CA (200 and 400 mg/kg body weight/day for 21 days): (**a**) NO level, (**b**) MDA level, (**c**) intracellular ROS generation: isolated islet cells undergo staining with H_2_DCFDA and subsequent analysis via flow cytometry (higher dose of BPA was considered only). The values displayed in the representative flow cytometry data represent the intensity of DCF fluorescence for the entire cell population. (**d**) The accompanying bar graph illustrates quantitative information regarding the mean DCFDA. Data were presented as Mean ± SEM (n = 3, for each sample isolated islets were pooled from two animals). CA (200 and 400 mg/kg body weight/day for 21 days) supplementation further harmonize BPA (10 and 100 µg/kg body weight for 21 days)-induced alteration in antioxidant parameters: (**e**) SOD activity (**f**), CAT activity (**g**), GSH level, and (**h**) glutathione peroxidise (GPx) activity. Except intracellular ROS, all data were presented as Mean ± SEM (n = 6). Normality of data was tested by Shapiro–Wilk test. Significance level based on one-way ANOVA, p < 0.05. Significance level based on Tukey’s post hoc test *p < 0.05, ** p < 0.01, ***p < 0.001, ****p < 0.0001, *ns* not significant.

Extensive research demonstrates that BPA disrupts pancreatic β-cell and α-cell functionality, leading to impaired glucose metabolism and insulin resistance, which are key factors in the development of metabolic disorders like diabetes^30^. Additionally, BPA-induced oxidative stress exacerbates insulin resistance, impairs pancreatic β-cell function, and promotes inflammation and tissue damage through lipid peroxidation and increased ROS production^30^. To assess the impact of BPA exposure on oxidative and nitrosative stress in islet cell lysates, we measured two key biomarkers: lipid peroxidation and nitric oxide production. Malondialdehyde (MDA) is the predominant and extensively researched product of lipid peroxidation, commonly employed as a biomarker to quantify oxidative stress across various biological samples^31^. This prompted us to include MDA measurements in our experimental setup. Additionally, we examined the role of reactive nitrogen species produced by nitric oxide (NO) in oxidative stress-induced cellular damage. Our analysis revealed a significant increase in MDA levels and NO generation (p < 0.0001) in the islet cell lysates of mice exposed to BPA at both doses (10 and 100 µg/kg body weight per day for 21 days) when compared to the control mice (Fig. 5a,b). However, the supplementation of CA was observed to substantially reverse these alterations, with the 400 mg/kg concentration displaying the highest level of effectiveness (p < 0.0001). In continuation, we further measured ROS generation in islet cells of BPA exposed mice (only higher dose was considered) and obtained BPA-induced increment in the ROS generation (Fig. 5c,d). Likewise, CA supplementation significantly blunted BPA-induced ROS generation (low dose: p < 0.001, high dose: p < 0.0001).

Figure 5e,f demonstrate that antioxidant enzymes, such as superoxide dismutase (SOD) and catalase (CAT) activity, were also decreased in the islet cell lysate of BPA-treated mice when compared to the control group (p < 0.0001). However, the administration of CA gradually increased the activity of these antioxidant enzymes in a concentration-dependent manner. The most significant enhancement in enzymatic activity was observed in the group supplemented with CA at the dose of 400 mg/kg (p < 0.0001).

In Fig. 5g,h, it is evident that the administration of BPA at two different doses led to a significant reduction in the levels of GSH and increase in the activity of GPx in islet cell lysate. Notably, both BPA doses (10 and 100 µg/kg) resulted in substantial reduction in GSH level and increment in GPx activity when compared to the control mice (p < 0.0001). As anticipated, the supplementation of CA at the higher dose effectively restored the GSH content and activity of GPx to a large extent (p < 0.0001).

### Protective efficacy of CA on BPA mediated impaired level of inflammatory cytokines and c-reactive protein

**Figure 6:**
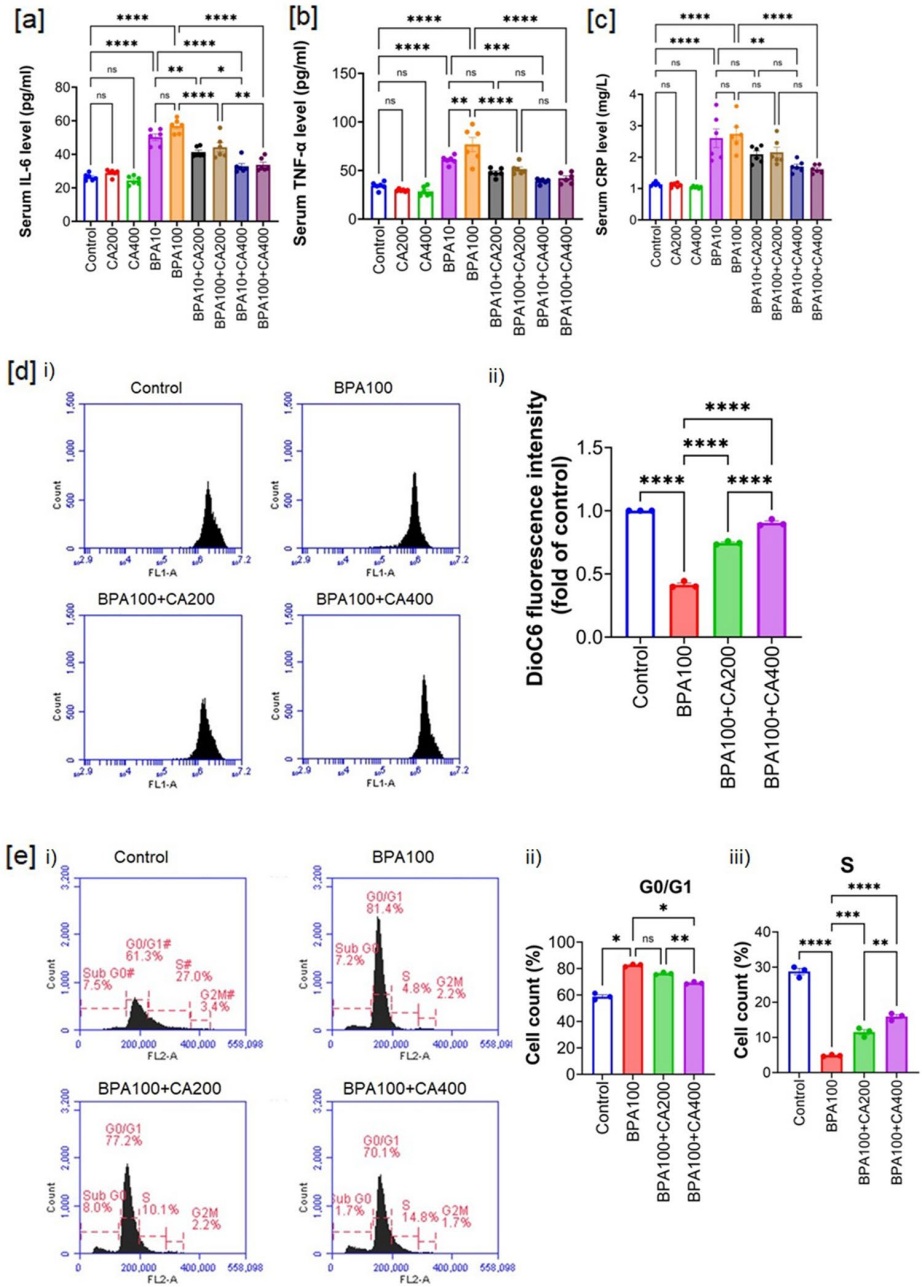
Impact of CA supplementation on heightened inflammation, loss of mitochondrial membrane potential (MMP) and impaired cell cycle caused by BPA. Serum level of (**a**) IL-6, (**b**) TNF-α and (**c**) c-reactive protein (CRP) in BPA (10 and 100 µg/kg body weight for 21 days) treated mice with or without supplementation of CA (200 and 400 mg/kg body weight/day for 21 days). Data were presented as mean ± SEM (n = 6). Effect of CA supplementation (200 and 400 mg/kg body weight/day for 21 days) on BPA (100 µg/kg body weight for 21 days)-mediated MMP loss and impaired cell cycle. (**d**) i) MMP loss measured by flow cytometry using the DiOC6 fluorescent probe ii) mean fluorescence intensity (as fold of control) (mean ± SEM, n = 3, for each sample isolated islets were pooled from two animals) of traces shown in (**d**) i). (**e**) i) flow cytometry analysis of cell cycle progression determined using propidium iodide (PI), ii) and iii) % of cells in G0/G1 and S, respectively (mean ± SEM, n = 3, for each sample isolated islets were pooled from two animals). Normality of data was tested by Shapiro–Wilk test. Significance level based on one-way ANOVA, p < 0.05. Significance level based on Tukey’s post hoc test *p < 0.05, ** p < 0.01, ***p < 0.001, ****p < 0.0001, *ns* not significant.

Elevated levels of pro-inflammatory cytokines in response to BPA exposure can trigger a stress response in pancreatic islets, leading to functional impairment^7^. Inflammatory cytokines such as tumor necrosis factor-alpha (TNF-α), and interleukin-6 (IL-6) can impair pancreatic β-cell function, leading to decreased insulin secretion^32^. Thus, measuring cytokine levels provide insight into the extent of inflammation that may contribute to impaired islet function caused by BPA treatment. Further, inflammatory cytokines can induce apoptosis in pancreatic β-cells. Chronic exposure to BPA may promote β-cell apoptosis through inflammatory pathways^30^. Therefore, assessing cytokine levels also helps evaluate the extent of β-cell death and its contribution to impaired islet function following BPA treatment. To ascertain the impact of BPA on inflammatory responses in mice, we measured the levels of proinflammatory cytokines in the serum (Fig. 6a,b). The levels of TNF-α and IL-6 were notably elevated in the BPA-treated groups in a concentration-dependent manner (10 and 100 µg/kg body weight), in comparison to the control group (p < 0.0001). However, this increase in cytokine levels was significantly mitigated following supplementation with two graded doses of CA.

C-reactive protein (CRP) levels in the blood are typically low in healthy individuals but can significantly rise in response to inflammation and tissue injury^33^. Our study results demonstrated a marked increase in serum CRP levels in BPA-treated mice (p < 0.001), following a dose-dependent pattern. Notably, the supplementation of CA was effective in reversing these effects, bringing the CRP levels closer to control values in a concentration-dependent manner (Fig. 6c). CRP levels, indicating body-wide inflammation, might not directly reflect islet cell damage from BPA. However, CRP could still signal inflammation, potentially exacerbating conditions linked to islet dysfunction like insulin resistance and impaired glucose metabolism, suggesting BPA-induced inflammation may indirectly harm pancreatic islets over time.

### CA supplementation mitigates BPA-mediated altered mitochondrial membrane potential (MMP) and cell cycle

Elevated levels of oxidative stress can disrupt the equilibrium between mitochondrial fission and fusion, often resulting in mitochondrial fragmentation and subsequent dysfunction^34^. In our current investigation, we found that BPA influences both oxidative stress and proinflammatory cytokines. This prompted us to expand our study to evaluate the mitochondrial membrane potential and cell cycle of pancreatic islet cells in mice exposed to 100 μg/kg body weight of BPA, with or without concurrent CA supplementation. The flow cytometric analysis of mitochondrial membrane potential (MMP) in pancreatic islet cells, conducted using the cationic dye DiOC6, revealed a significant disturbance in MMP in mice treated with BPA (Fig. 6d). However, the supplementation of CA had a notable mitigating effect on the BPA-induced loss of MMP.

By monitoring the cell cycle, variations in the rate of proliferation among islet cells can be evaluated. Thus, to delineate that BPA exposure could potentially impact the proliferation of pancreatic β-cells, we assessed the cell cycle of pancreatic islet cells in BPA exposed mice. Further, observing alterations in the cell cycle, including shifts in the distribution of cells across phases like G1, S, G2, and M, can serve as indicators of disturbances in cellular proliferation instigated by BPA. Therefore, to evaluate the influence of BPA on the cell cycle, we employed flow cytometry to assess the proportions of SubG0, G0/G1-, S-, and G2/M phase of pancreatic islet cells. In contrast to the control group, the analysis of the cell cycle in pancreatic islet cells revealed a notable increase in the G0/G1 percentage accompanied by a simultaneous decrease in the S percentage after BPA administration (Fig. 6e). These results suggest that BPA accelerates the arrest of pancreatic islet cells at the transition from the G0/G1 phase to the S phase of the cell cycle. In the BPA-exposed group supplemented with CA, there was a significant reduction in the proportion of G0/G1 phase cells and a notable increase in the proportion of S phase cells. Taken together, we hypothesized that BPA exposure might disrupt the normal progression of the cell cycle in islet cells, potentially causing a delay or arrest in the G0/G1 phase. The observation that CA supplementation promotes the transition of these cells from G0/G1 to the S phase implies that CA may mitigate or counteract the effects of BPA-induced cell cycle disruption.

### Protective efficacy of CA on BPA-induced apoptosis and expression of Bcl_2_, Bax, cleaved-caspase-3 and caspase-9 in pancreatic islets

**Figure 7:**
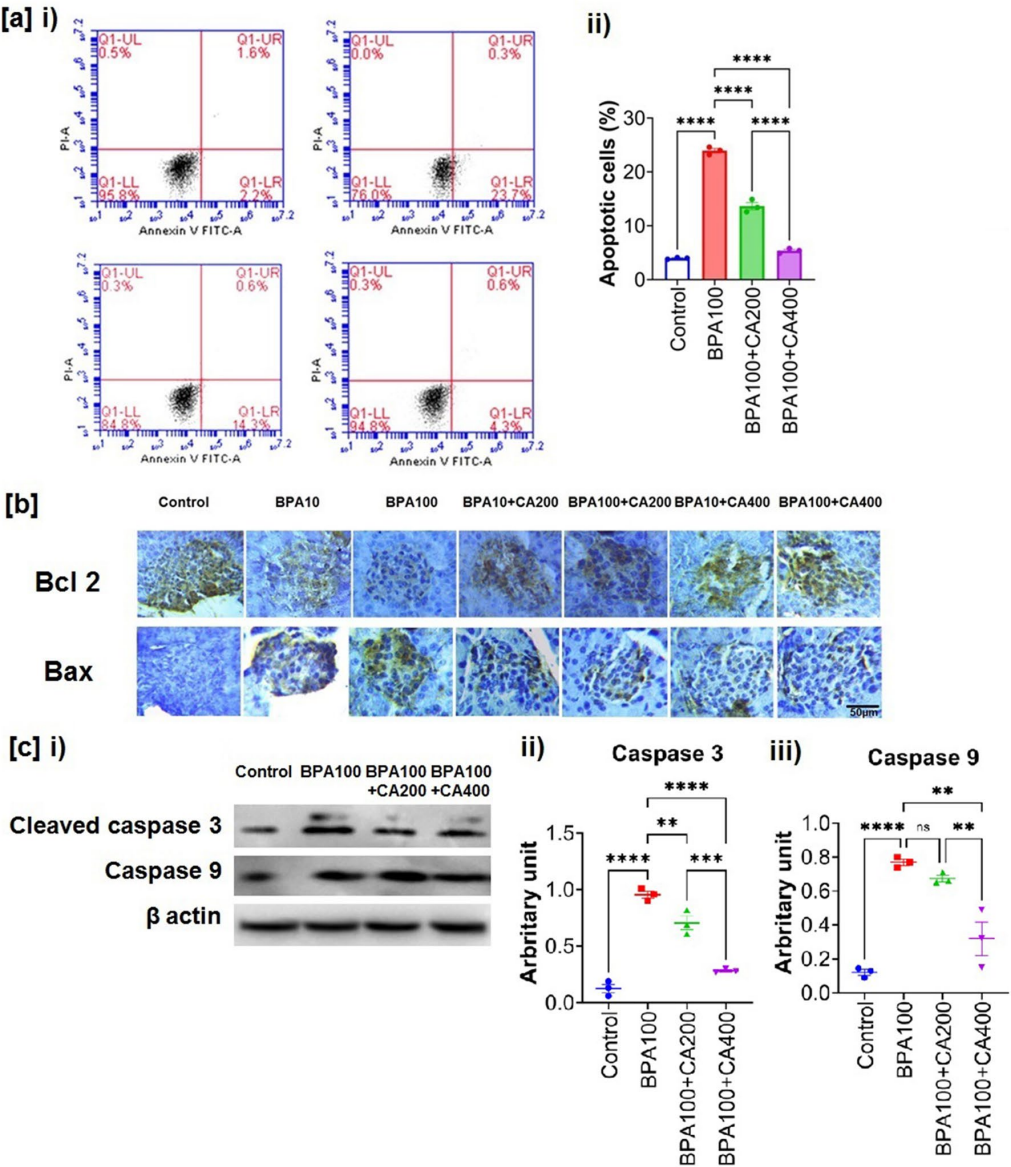
Protective role of CA against BPA-induced apoptosis of pancreatic islet cell and altered expression of Bcl2, Bax, cleaved caspase-3 and caspase-9. Effect of BPA (100 µg/kg body weight for 21 days) on apoptosis of pancreatic islet cells and expression of pro-apoptotic and anti-apoptotic markers with or without supplementation of ethanol extract of CA (200 and 400 mg/kg body weight/day for 21 days). (**a**) i) flow cytometry analysis of apoptosis determined using Annexin V/PI, ii) Apoptotic cells (mean ± SEM, n = 3, for each sample isolated islets were pooled from two animals) of traces shown in (**a**) i). (**b**) i) Representative photomicrograph of immunohischemical analysis of Bcl2 and Bax in pancreatic islets. Results are representative of six mice. Magnification ×200 and scale bar: 50 µm for all panels. (**c**) i) Western blot of cleaved caspase-3 and caspase 9, ii) and iii) Quantification of cleaved caspase-3 (17 kDa) and caspase 9 (mean ± SEM, n = 3, for each sample isolated islets were pooled from two animals). Normality of data was tested by Shapiro–Wilk test. Significance level based on one-way ANOVA, p < 0.05. Significance level based on Tukey’s post hoc test *p < 0.05, **p < 0.01, ***p < 0.001, ****p < 0.0001, *ns* not significant.

The mitochondrial membrane potential (ΔΨm) plays a crucial role in apoptosis^35^, with changes in ΔΨm known to precede caspase activation and cell death. Given BPA's demonstrated impact on mitochondrial membrane potential and the cell cycle in this study, we further investigated apoptosis, along with pro-apoptotic and anti-apoptotic proteins, in the current experimental setup. Flow cytometric analysis of pancreatic islet cells isolated from mice exposed to BPA revealed an increased number of apoptotic cells compared to the control group (Fig. 7a). However, the supplementation of CA at two different doses effectively mitigated the induction of apoptosis in pancreatic islets, with the higher dose of CA demonstrating superior efficacy.

The expression levels of pro-apoptotic markers (cleaved-caspase-3, caspase-9 and Bax) were observed to be up-regulated in mice treated with BPA compared to the control group (Fig. 7b,c, supplementary Figure S2). Conversely, the anti-apoptotic marker Bcl2 showed a significant decrease in pancreatic islets of BPA-treated mice (Fig. 7b). However, CA was found to finely regulate the BPA-induced imbalance between pro-apoptotic and anti-apoptotic markers, thereby rescuing pancreatic islet cells from BPA-induced toxicity and apoptosis.

## Discussion

A growing body of evidence has brought to light the potential role of endocrine-disrupting chemicals (EDCs), such as BPA, in the onset of type 2 diabetes mellitus. The study of EDC effects and the urgent need to counteract their impact using natural remedies have become pressing concerns. This research provides concrete experimental proof of ethanol extract of *Centella asiatica* (CA)’s ability to alleviate BPA-induced islet cell dysfunction and insulin resistance in mice by providing a hub that links inflammation and oxidative stress and apoptosis—well-documented factors contributing to tissue damage associated with hyperglycemia.

Earlier report indicates that male rats exposed to a daily dose of 10 µg/kg of BPA for 8 days experienced fasting hyperglycemia and glucose intolerance^36^. In accordance with this study, elevated fasting blood glucose (FBG) levels were likewise observed in BPA-exposed mice, alongside the presence of glucose intolerance. If not managed, persistent hyperglycemia can lead to deterioration in glucose tolerance, ultimately culminating in the development of type 2 diabetes mellitus. The Oral Glucose Tolerance Test (OGTT) is the most commonly employed diagnostic test in clinical practice for identifying glucose intolerance and diabetes mellitus^37^. Higher AUC of oral glucose tolerance in BPA-treated mice revealed impaired insulin sensitivity in the current study. The CA, rich in various triterpenes like asiatic acid, asiaticoside, and madecassic acid (as evidenced by HPLC analysis of CA in this current investigation), demonstrated an improvement in BPA-induced impaired fasting blood glucose (FBG) and oral glucose tolerance. This suggests its beneficial impact in a context where BPA exerts diabetogenic effects. Insulin resistance initiates and advances prior to the development of hyperglycemia, and it lingers for a substantial duration before leading to reduced glucose uptake in specific tissues^38^.

In this study, mice treated with BPA showed increased levels of plasma insulin and c-peptide. C-peptide, a proinsulin fragment released from pancreatic β cells, mirrors endogenous insulin production, making it a valuable marker for assessing pancreatic β cell function alongside insulin. In this experimental context, BPA was found to enhance the release of both insulin and c-peptide. This observation indicates a diminished capacity of islet cells to manage glucose load, which in turn triggers the onset of insulin resistance. Interestingly, supplementation of CA simultaneously with BPA significantly controlled the exaggerated release of both insulin and c-peptide.

In this study, HOMA-IR and HOMA-β were calculated to assess peripheral insulin resistance and β-cell function, respectively^39,40^. BPA-exposed mice exhibited elevated HOMA-IR, indicating reduced insulin sensitivity, while lower HOMA-β scores suggested β-cell damage, revealing the strain on pancreatic cells combating hyperglycemia due to BPA exposure. Nonetheless, the administration of two different doses of CA had a substantial effect on the HOMA-IR index and HOMA-β scores that were disrupted by BPA, steering them towards the enhancement of pancreatic beta cell function.

The study confirms a decline in insulin-positive cells and an increase in vacuolization in pancreatic islets of BPA-treated mice, observed through immunohistochemical and haematoxylin–eosin staining, respectively. Furthermore, the study foresaw these outcomes through the augmented median diameter of pancreatic islets in BPA-exposed mice. It is postulated that this increase in median islet diameter anticipates potential damage to islet cells, leading to a reduction in insulin expression and an elevation in glucose load induced by BPA exposure. However, *Centella asiatica* (CA) supplementation exhibits protective effects, enhancing insulin expression and reducing vacuolization and median islet diameter. Typically, individuals exhibiting insulin resistance tend to display elevated triglyceride levels and lower levels of HDL-C. Previous research has underscored the significance of lipid ratios as practical markers for assessing insulin resistance^41^. The dyslipidemia induced by BPA treatment reinforces the development of insulin resistance, a phenomenon potentially mitigated by CA treatment.

Numerous factors and mechanisms contribute to the reduction in beta-cell mass and function, and understanding these processes is pivotal for the development of novel therapeutic approaches aimed at enhancing beta-cell function^42^. It has been demonstrated that oxidative stress plays a role in mediating many of these processes. Pancreatic beta-cells are characterized by having a lower antioxidant capacity compared to most other tissues^43^. The activity of superoxide dismutase (SOD), which eliminate ·O^2−^, are approximately 50% lower in beta-cells, and the levels of enzymes that inactivate H_2_O_2_, such as catalase (CAT) and glutathione peroxidase (GPx), are only about 1% of their respective levels in the liver. These inherent antioxidant properties of islets allow for regulated insulin secretion mediated by reactive oxygen species. However, when oxidative stress becomes amplified and persistent, it can lead to impaired insulin secretion and ultimately result in damage and destruction of beta-cells^44^.

The development of insulin resistance is significantly impacted by inflammation, and in this study, BPA-induced insulin resistance correlates with increased TNF-α and IL6 production, suggesting a connection between inflammation, diabetes, and pancreatic islet dysfunction^45^. Nonetheless, the addition of CA was demonstrated to mitigate the dose-dependent increase in the production of crucial pro-inflammatory cytokines, namely TNF-α and IL-6, induced by BPA. Consequently, the anti-inflammatory attributes of CA, as observed in this study, may serve as a mechanism to safeguard pancreatic islets from BPA-induced cytokine-related harm. This could hold therapeutic significance in preventing the advancement of islet cell dysfunction. Moreover, previous reports have indicated a significant association between c-reactive protein (CRP), a sensitive marker of systemic inflammation, and type 2 diabetes mellitus^46^. In the present context, the elevated CRP levels in BPA-treated mice suggest a systemic state of inflammation and islet cell dysfunction, which was effectively mitigated by the administration of two different doses of CA (200 and 400 mg/kg body weight).

Mitochondria are susceptible to oxidative stress and prolonged reactive oxygen species (ROS) production can alter their structure, triggering mitochondrial transition pore opening and loss of mitochondrial membrane potential (MMP)^47^. Further, a decline in membrane potential serves as an early indicator of apoptosis. BPA exposure in pancreatic islets precedes apoptosis by reducing mitochondrial membrane potential (MMP), confirmed via Annexin V-PI flow cytometry. Additionally, BPA exhibited a noteworthy effect on the cell cycle progression of pancreatic islet cells by arresting the cell cycle at the G0/G1 phase, consequently impeding progression into the S phase. Therefore, it is crucial to emphasize that BPA triggers apoptosis in pancreatic islet cells, leading to onset of islet cell dysfunction and disruptions in glucose homeostasis. Supplementing CA to mice exposed to BPA not only demonstrated a beneficial impact but also mitigated BPA-induced MMP loss, elevated apoptosis, and impaired cell cycle, as observed in the current study. This highlights CA as a promising option in countering the diabetogenic effects of BPA. In this study, BPA reduced Bcl-2 levels while elevating Bax, promoting apoptosis. This imbalance in the Bcl-2 and Bax ratio, favoring Bax, suggests a propensity for apoptosis induced by BPA. The supplementation of CA counteracts this imbalance, tipping the scale in favor of Bcl-2. This restoration in the balance between Bcl-2 and Bax, facilitated by CA supplementation, underscores the protective potential of CA against the BPA-induced apoptosis of pancreatic islet cells. Furthermore, the heightened levels of pro-inflammatory cytokines, as observed in this study and corroborated by findings in another study by Cnop et al.^48^, could potentially contribute to β-cell apoptosis in T2DM. However, the extent to which these pathways overlap has been a subject of questioning in the study by Cnop et al.^48^.

Moreover, pancreatic β-cell apoptosis is facilitated by caspase-3 and caspase-9, crucial mediators in the intrinsic pathway of apoptosis, with caspase-3 serving as a pivotal point in this pathway^49^. Increased cleaved caspase-3 and caspase-9 in pancreatic islets confirm apoptosis in BPA-exposed mice. CA protects islets by enhancing Bcl2 levels, impeding death signaling and reducing pro-apoptotic proteins like Bax and caspases. BPA disrupts apoptotic balance, leading to β-cell death, but CA rescues β-cells significantly from BPA damage. In conclusion, CA supplementation emerges as a robust protective measure against BPA-induced toxicity in the endocrine pancreas, positioning itself as a promising intervention strategy to counteract the diabetogenic effects of BPA.

## Materials and methods

### Plant material

We obtained *Centella asiatica* leaves from a local marketplace in Kolkata, West Bengal, India. After authentication by Botanical Survey of India, fresh and fully mature leaves underwent a meticulous washing process with running water. Dried leaf powder was prepared according to the method outlined by Saranya et al.^50^. To prepare the crude extract, *Centella asiatica* leaf powder underwent extraction using a 95% ethanol solvent, undergoing maceration at room temperature for 72 h. The resulting extract was then manually squeezed through a thin cloth and filtered using Whatman filter paper no. 1. Employing a rotary evaporator, the filtrate was concentrated under reduced pressure at temperatures below 50 °C. The concentrated extracts were subsequently poured into Pyrex glass petri dishes (90 × 15 mm) to facilitate complete solvent evaporation^50^. The resulting crude extract was either suspended in distilled water for immediate use or stored in the refrigerator at 4 °C for future studies.

### HPLC analysis of the plant extract

The plant extract was analyzed using chromatographic technique following the protocol outlined by Monton et al.^51^. High-performance liquid chromatography (HPLC) analyses were conducted employing the Dionex Ultimate 3000 liquid chromatograph from Germany. The system featured a four-solvent delivery system quaternary pump (LPG 3400 SD), along with a diode array detector (DAD 3000) equipped with a 5-cm flow cell and a manual sample injection valve fitted with a 20 µl loop. Data processing was performed using Chromeleon 6.8 syringe management. For the separation process, a reversed-phase Acclaim TM 120 C18 column with a particle size of 5 µm and dimensions of 4.6 × 250 mm was utilized. To prepare working solutions, a stock solution (1 mg/ml) containing asiatic acid, madecassic acid, and asiaticoside was diluted with HPLC-grade methanol. This dilution aimed to achieve concentrations of 60, 50, 40, 30, 20, and 10 µg/ml for each respective compound. Before injecting the standard and working solutions, the mobile phase underwent degassing, and both types of solutions were filtered through a 0.45 µm PVDF-syringe filter.

### Experimental animals

The present study was conducted on male Swiss mice (body weight between 25 and 30 g) considering all ethical standards set by the Institutional Animal Ethics Committee (IAEC) of Serampore College (registered under CPCSEA, Government of India). The animals were housed in a well-ventilated facility with suitable lighting conditions and were kept at a consistent temperature of 24 ± 3 °C. Prior to initiating the experiments, a 7-day acclimatization period was granted to the mice. During this period, the mice were given unrestricted access to water and provided with a standard diet, adhering to the guidelines set forth by Mukherjee et al.^52^. The selection of male animals for this study was influenced by research findings that demonstrated a higher concentration of serum BPA in male animals compared to females, even when exposed to equivalent levels of the compound^9^. The methods utilized in this study underwent review and received approval from the Institutional Animal Ethics Committee (IAEC) of Serampore College, with the assigned approval number 21/P/S/SC/IAEC/2019. Additionally, all animal experiments were conducted in accordance with the ARRIVE guidelines.

### Acute toxicity study

An acute oral toxicity study of *Centella asiatica* (CA) was carried out in adherence to the guidelines outlined by the Organization for Economic Co-operation and Development (OECD) in 2001^53^. Seven different doses (125 mg/kg, 250 mg/kg, 500 mg/kg, 1000 mg/kg, 2000 mg/kg, 3000 mg/kg and 4000 mg/kg body weight) were used for acute toxicity study and control group was given normal saline (n = 3 for each group). The animals were closely monitored for their appearance and observed for any behavioral changes during the initial 4 h. Subsequent observations were conducted periodically over the next 24 h and then daily for a total period of 14 days. Each cage received the necessary amount of food and water daily. Changes in food and water intake, as well as fluctuations in body weight, were meticulously noted alongside ongoing cage-side observations.

### Experimental design

54 Swiss mice (4–6 weeks old) were randomly divided into nine groups (n = 6): Control (vehicle treated), CA200 (CA: 200 mg/kg body weight), CA400 (CA: 400 mg/kg body weight), BPA10 (10 µg/kg body weight), BPA100 (100 µg/kg body weight), BPA10 + CA200 (BPA: 10 µg/kg body weight, CA: 200 mg/kg body weight), BPA10 + CA400 (BPA: 10 µg/kg body weight, CA: 400 mg/kg body weight), BPA100 + CA200 (BPA: 100 µg/kg body weight, CA: 200 mg/kg body weight), BPA100 + CA400 (BPA: 100 µg/kg body weight, CA: 400 mg/kg body weight). BPA was dissolved in olive oil and administered via intraperitoneal injection twice daily, precisely at 9:00 a.m. and 2:00 p.m., consistently for duration of 21 days. The dosage of BPA was determined using the methodology outlined by Alonso-Magdalena et al.^54^, with specific modifications. The concentration of the ethanol extract of *Centella asiatica* (CA) was selected in accordance with the study conducted by Giribabu et al.^55^, with slight modifications, and considering preliminary findings from our own research.

### Determination of fasting blood glucose (FBG) and oral glucose tolerance test (OGTT)

Following the completion of the experimental period (21 days), mice underwent a 12-h fasting period for the measurement of fasting blood glucose (FBG) and oral glucose tolerance test (OGTT). The next morning, blood samples were collected from the tail vein. After the initial blood sample collection, all animals received 1 g of glucose per kg of body weight through oral gavage. Subsequent blood samples were taken at 30-min intervals up to 120 min following the glucose administration. Glucose oxidase enzyme kit (E. Merck, India) was used for measurement of blood glucose level.

### Preparation of serum and plasma

Upon completing the blood collection for fasting blood glucose (FBG) and oral glucose tolerance test (OGTT), mice were sedated with pentobarbitone sodium at a dose of 60 mg per kilogram of body weight through intraperitoneal injection. Subsequently, blood samples were obtained by cardiac puncture and divided into two separate, sterile centrifuge tubes. One tube contained EDTA to prevent coagulation, while the other did not. The samples were then processed to separate the plasma and serum components. Following separation, these samples were stored at a temperature of − 80 °C until ready for use in subsequent biochemical tests.

### Measurement of insulin, c-peptide and calculation of different indexes

We employed an ELISA kit (Raybiotech in Norcross, GA) to measure the levels of c-peptide and fasting plasma insulin (Fins). Following this, we calculated HOMA-IR (Homeostatic Model Assessment of Insulin Resistance) and HOMA-β (Homeostatic Model Assessment of β Cell Function) using the following formulas:

$${\text{HOMA-}}\mathrm{IR }=[\mathrm{FBG }({\text{mmol}}/\mathrm{L \times Fins}(\mathrm{\mu \,IU}/{\text{L}})]/22.5$$

$${\text{HOMA-}}\upbeta = 20 \times \mathrm{ Fins }(\mathrm{\mu IU}/{\text{L}}) /[{\text{FBG}}({\text{mmol}}/{\text{L}})-3.5]$$

### Measurement of TNF-α and IL-6

We utilized the ELISA kit from Raybiotech (Norcross, GA, USA) to assess the concentrations of serum TNF-α and IL-6. Duplicate analyses were performed for each sample. The intra-assay variance for TNF-α and IL-6 was 6.7% and 5.5%, respectively. Furthermore, serum CRP levels were assessed utilizing an Invitrogen (Thermo Fisher Scientific, USA) mouse CRP ELISA kit. For CRP, the intra-assay variation was measured at 3.4%. To mitigate inter-assay variance, all samples were processed simultaneously.

### Measurement of lipid profile parameters

Total cholesterol (TC), serum triglycerides, and high-density lipoprotein (HDL) levels were determined using biochemical kits obtained from ERBA diagnostics (Mumbai, India), while very low-density lipoprotein (VLDL) was computed as triglyceride/5. Additionally, low-density lipoprotein (LDL) was calculated using the formula: LDL = total cholesterol − (HDL + VLDL).

### Isolation of islet cells

Following blood collection, the mice were euthanized through cervical dislocation, and pancreatic islets were isolated using the collagenase digestion method as outlined by Neuman et al.^56^. The purity of the isolated islets was confirmed through dithizone staining. To obtain a single-cell suspension, the primary islets were mechanically dissociated by pipetting for 5 min at 37 °C in a 0.25% trypsin–EDTA solution. The cells were gently pipetted, cleaned, and then re-suspended in phosphate-buffered saline (PBS) for subsequent applications.

### Estimation of islet cell protein content

Islet cell protein content was determined according to the method developed by Lowry et al.^57^ using bovine serum albumin as standard.

### Measurement of oxidative stress parameters

Thiobarbituric-acid-reactive substances (TBARS) was measured employing the method outlined by Wills^58^ and used as a measure of lipid peroxidation in isolated islet cells. The results were quantified and expressed as nanomoles of malondialdehyde (MDA) per milligram of protein. Nitrobluetetrazolium (NBT) was utilized to measure superoxide dismutase (SOD) activity, following the protocol described by Sun et al.^59^. Catalase activity was quantified using the spectrophotometric method detailed by Aebi^60^, involving the decomposition of H_2_O_2_ at 240 nm. The level of reduced glutathione (GSH) in islet cell lysate was determined using Ellman's method with 5,5ʹ-dithiobis-2-nitrobenzoic acid (DTNB)^61^. The activity of glutathione peroxidase (GPx) was assessed following the method outlined by Paglia and Valentine^62^.

### Flow cytometric analysis of intracellular reactive oxygen species (ROS), mitochondrial membrane potential (MMP), cell cycle and apoptosis

Intracellular ROS levels of isolated pancreatic islet cells were measured by flow cytometry (BD Accuri Flow Cytometer) using 2,7-dichlorofluorescein diacetate (DCFDA) fluorescence dye^63^. Following isolation, islet cells were treated with 1 μM DCFDA and incubated at 37 °C for 30 min in the dark. After staining, the cells were passed through a 40 μm cell strainer to eliminate cell clumps.

The mitochondrial membrane potential was assessed using the fluorochrome stain DiOC6 (Molecular Probes). Isolated islet cells were loaded with 40 nM DiOC6 for 30 min and then analyzed using a BD Accuri Flow Cytometer.

For cell cycle analysis, cells were fixed with chilled 70% ethanol overnight at 4 °C. Cells were washed twice with PBS at 1200 rpm for 5 min to remove ethanol and finally cells were incubated propidium iodide in dark for 30 min and then acquired on a BD Accuri Flow Cytometer with excitation at 488 nm and emission was collected by 585/40 nm band pass filter.

Apoptosis in isolated islet cells was evaluated utilizing the Annexin V fluorescent probe. This protein binds to phosphatidylserine residues exposed on the cell surface of apoptotic cells. Flow cytometry was employed to monitor apoptosis, employing Annexin V and Propidium Iodide with a 488 nm laser and detecting fluorescence in FL1 (530/30) and FL2 (585/40) channels.

### Western blot

Following the lysis of isolated islet cells with RIPA buffer, the protein content was determined using the method described by Lowry et al.^57^. Subsequently, equivalent quantities of proteins were separated using sodium dodecyl sulfate–polyacrylamide gel electrophoresis (SDS-PAGE) gels and then transferred to polyvinylidene fluoride membranes. To block non-specific binding sites on the membranes, 50 g/l nonfat milk in TBST (20 mMTris-Hcl, pH 7.5, 150 mMNaCl, 1 g/l Tween20) washing buffer was applied for 2 h at room temperature. The membranes were then incubated overnight at 4 °C with specific primary antibodies against cleaved-caspase 3 (1:5000, Abcam, Cambridge, United Kingdom) and caspase-9 (1:2000, Abcam, Cambridge, United Kingdom). After necessary washes, the membranes were incubated with peroxidase-labeled secondary antibodies at a dilution of 1:20,000 (Abcam, Cambridge, United Kingdom) at room temperature for 1 h. Immunoreactivity was detected using the ECL detection kit (Bio Rad Laboratories India Pvt Ltd) through chemiluminescence. Gel-Pro Analyzer 6.0 application was employed for densitometry to measure band intensity in relation to the β-actin bands (Media Cybernetics, Silver Spring, MD, USA).

### Preparation of permanent slides and immunohistochemical analysis

In preparation for histopathological and immunohistochemical analysis, we prepared permanent pancreas slides. Pancreatic tissue from the gastro-splenic region was selectively extracted and fixed using formol fixative. Subsequently, paraffin blocks were sliced into 4–5 µm-thick sections using a rotary microtome, and standard microscopic slides were produced. These slides were then subjected to examination under light microscopy (Carl Zeiss, Primostar model) following staining with haematoxylin and eosin^64^. Vacuolization of pancreatic islets was quantified using vacuolization score: > 2 vacuoles/100 μm islet diameter—1, > 4 vacuoles/100 μm islet diameter—2, > 8 vacuoles/100 μm islet diameter—3.

For immunohistochemical analysis, 5-µm thick sections of pancreatic tissue were initially subjected to deparaffinization and rehydration. Following this step, the sections were boiled in 250 ml of an antigen retrieval buffer (containing 10 mM sodium citrate, pH 6.0) for 30 min. After cooling and intermediate washes, the slides underwent a double 10-min wash in TBS with 0.025% Triton X-100 (TBST) buffer, with gentle agitation. Subsequently, the sections were incubated for 2 h at room temperature in a solution comprising 10% serum and 1% BSA in TBS. Overnight incubation at 4 °C followed with specific primary antibodies targeting insulin (1:64000, Abcam, Cambridge, United Kingdom), Bcl2 (1:100, Abcam, Cambridge, United Kingdom), and Bax (1:50, Abcam, Cambridge, United Kingdom). The next day, thorough washing was conducted, and the sections were exposed to 0.3% hydrogen peroxide in TBS for 15 min. Subsequently, the slides were incubated with a secondary antibody, specifically goat anti-rabbit biotin-conjugated (Abcam, Cambridge, United Kingdom), at a dilution of 1:500 for 1 h at room temperature. Following this, the sections were stained with DAB chromogen (Abcam, Cambridge, United Kingdom) for 10 min. After a 5-min rinse in running tap water, the sections underwent counterstaining with hematoxylin, dehydration in increasing alcohol concentrations, mounting, and final assessment under a light microscope. Immunohistochemical analysis of insulin positive cells was quantified by Image J (NIH, Bethesda, MD, USA) and expressed as integrated density (pixel intensity).

#### Statistical analysis

Statistical analysis was performed using GraphPad Prism 10.0. The data was expressed as mean ± SEM (standard error of mean). The normality of the data was evaluated using Shapiro–Wilk test. To identify significant differences between the groups, a one-way ANOVA test was applied. Following this, Tukey's multiple comparison test was utilized to determine whether the scores for the individual groups exhibited significant distinctions. A significance level of p < 0.05 was employed to ascertain the presence of statistically significant differences.

## Supplementary Information

Supplementary Information.

## Data Availability

Data will be made available on request (Contact: Dr. Sandip Mukherjee, Email ID: sandip@seramporecollege.ac.in).

## References

[1] Sun, H. *et al.* Global, regional and country-level diabetes prevalence estimates for 2021 and projections for 2045. *Diabetes Res. Clin. Pract.* **183**, 109119. 10.1016/j.diabres.2021.109119 (2022). 34879977 10.1016/j.diabres.2021.109119 PMC11057359

[2] Bragg, F. *et al.* Association between diabetes and cause-specific mortality in rural and urban areas of China. *JAMA* **317**(3), 280–289. 10.1001/jama.2016.19720 (2017). 28114552 10.1001/jama.2016.19720 PMC6520233

[3] Policardo, L. Effect of diabetes on hospitalization for ischemic stroke and related in-hospital mortality: A study in Tuscany, Italy, over years 2004–2011. *Diabetes Metab. Res. Rev.* **31**(3), 280–286. 10.1002/dmrr.2607 (2015). 25255901 10.1002/dmrr.2607

[4] Chen, H., Chen, G., Zheng, X. & Guo, Y. Contribution of specific diseases and injuries to changes in health adjusted life expectancy in 187 countries from 1990 to 2013: Retrospective observational study. *BMJ (Clin. Res. Ed)* **364**, l969. 10.1136/bmj.l969 (2019). 10.1136/bmj.l969 PMC6435998 30917970

[5] Lin, J. Y. & Yin, R. X. Exposure to endocrine-disrupting chemicals and type 2 diabetes mellitus in later life. *Expo Health* **15**, 199–229. 10.1007/s12403-022-00486-0 (2023).

[6] Sargis, R. M. & Simmons, R. A. Environmental neglect: Endocrine disruptors as underappreciated but potentially modifiable diabetes risk factors. *Diabetologia* **62**(10), 1811–1822. 10.1007/s00125-019-4940-z (2019). 31451869 10.1007/s00125-019-4940-z PMC7462102

[7] Banerjee, O. *et al.* Molecular dissection of cellular response of pancreatic islet cells to Bisphenol-A (BPA): A comprehensive review. *Biochem. Pharmacol.* **201**, 115068. 10.1016/j.bcp.2022.115068 (2022). 35504317 10.1016/j.bcp.2022.115068

[8] Manzoor, M. F. *et al.* An insight into bisphenol A, food exposure and its adverse effects on health: A review. *Front. Nutr.* **9**, 1047827. 10.3389/fnut.2022.1047827 (2022). 36407508 10.3389/fnut.2022.1047827 PMC9671506

[9] Caporossi, L. & Papaleo, B. Bisphenol A and metabolic diseases: Challenges for occupational medicine. *Int. J. Environ. Res. Public Health* **14**(9), 959. 10.3390/ijerph14090959 (2017). 28841159 10.3390/ijerph14090959 PMC5615496

[10] Rohm, T. V., Meier, D. T., Olefsky, J. M. & Donath, M. Y. Inflammation in obesity, diabetes, and related disorders. *Immunity* **55**(1), 31–55. 10.1016/j.immuni.2021.12.013 (2022). 35021057 10.1016/j.immuni.2021.12.013 PMC8773457

[11] Moon, M. K. *et al.* Long-term oral exposure to Bisphenol A induces glucose intolerance and insulin resistance. *J. Endocrinol.* **226**(1), 35–42. 10.1530/JOE-14-0714 (2015). 25972359 10.1530/JOE-14-0714

[12] Puttabyatappa, M. *et al.* Developmental programming: Changes in mediators of insulin sensitivity in prenatal bisphenol A-treated female sheep. *Reprod. Toxicol.* **85**, 110–122. 10.1016/j.reprotox.2019.03.002 (2019). 30853570 10.1016/j.reprotox.2019.03.002 PMC6443435

[13] Beler, M. *et al.* Bisphenol A reveals its obesogenic effects through disrupting glucose tolerance, oxidant-antioxidant balance, and modulating inflammatory cytokines and fibroblast growth factor in zebrafish. *Toxicol. Ind. Health* **38**(1), 19–28. 10.1177/07482337211054372 (2022). 35090367 10.1177/07482337211054372

[14] Zhao, Y. *et al.* Effect of *Centella asiatica* on oxidative stress and lipid metabolism in hyperlipidemic animal models. *Oxid. Med. Cell Longev.* **2014**, 154295. 10.1155/2014/154295 (2014). 24829618 10.1155/2014/154295 PMC4009232

[15] Gohil, K. J., Patel, J. A. & Gajjar, A. K. Pharmacological review on *Centella asiatica*: A potential herbal cure-all. *Indian J Pharm Sci.* **72**(5), 546–556. 10.4103/0250-474X.78519 (2010). 21694984 10.4103/0250-474X.78519 PMC3116297

[16] Wong, J. H., Barron, A. M. & Abdullah, J. M. Mitoprotective effects of *Centella asiatica* (L.) Urb: Anti-inflammatory and neuroprotective opportunities in neurodegenerative disease. *Front. Pharmacol.* **12**, 687935. 10.3389/fphar.2021.687935 (2021). 34267660 10.3389/fphar.2021.687935 PMC8275827

[17] Oyenihi, A. B., Chegou, N. N., Oguntibeju, O. O. & Masola, B. *Centella asiatica* enhances hepatic antioxidant status and regulates hepatic inflammatory cytokines in type 2 diabetic rats. *Pharm. Biol.* **55**(1), 1671–1678. 10.1080/13880209.2017.1318293 (2017). 28447512 10.1080/13880209.2017.1318293 PMC6130484

[18] James, J. T. & Dubery, I. A. Pentacyclic triterpenoids from the medicinal herb, *Centella asiatica* (L.) Urban. *Molecules (Basel, Switzerland)* **14**(10), 3922–3941. 10.3390/molecules14103922 (2009). 19924039 10.3390/molecules14103922 PMC6255425

[19] Cao, W. *et al.* Madecassoside suppresses LPS-induced TNF-alpha production in cardiomyocytes through inhibition of ERK, p38, and NF-kappaB activity. *Int. Immunopharmacol.* **10**(7), 723–729. 10.1016/j.intimp.2010.03.015 (2010). 20381648 10.1016/j.intimp.2010.03.015

[20] Ramachandran, V. & Saravanan, R. Efficacy of asiatic acid, a pentacyclic triterpene on attenuating the key enzymes activities of carbohydrate metabolism in streptozotocin-induced diabetic rats. *Phytomed* **20**(3–4), 230–236. 10.1016/j.phymed.2012.09.023 (2013). 10.1016/j.phymed.2012.09.023 23102509

[21] Ramachandran, V. & Saravanan, R. Glucose uptake through translocation and activation of GLUT4 in PI3K/Akt signaling pathway by asiatic acid in diabetic rats. *Human Exp. Toxicol.* **34**(9), 884–893. 10.1177/0960327114561663 (2015). 10.1177/0960327114561663 26286522

[22] Yousef, H. *et al.* Inflammation, oxidative stress and mitochondrial dysfunction in the progression of type II diabetes mellitus with coexisting hypertension. *Front. Endocrinol.* **14**, 1173402. 10.3389/fendo.2023.1173402 (2023). 10.3389/fendo.2023.1173402 PMC10296202 37383391

[23] Sun, B. *et al.* Therapeutic potential of *Centella asiatica* and its triterpenes: A review. *Front. Pharmacol.* **11**, 568032. 10.3389/fphar.2020.568032 (2020). 33013406 10.3389/fphar.2020.568032 PMC7498642

[24] Maddison, L. A., Joest, K. E., Kammeyer, R. M. & Chen, W. Skeletal muscle insulin resistance in zebrafish induces alterations in β-cell number and glucose tolerance in an age- and diet-dependent manner. *Am. J. Physiol. Endocrinol. Metab.* **308**(8), E662–E669. 10.1152/ajpendo.00441.2014 (2015). 25670827 10.1152/ajpendo.00441.2014 PMC4398831

[25] Yoon, H., Jeon, D. J., Park, C. E., You, H. S. & Moon, A. E. Relationship between homeostasis model assessment of insulin resistance and beta cell function and serum 25-hydroxyvitamin D in non-diabetic Korean adults. *J. Clin. Biochem. Nutr.* **59**(2), 139–144. 10.3164/jcbn.15-143 (2016). 27698542 10.3164/jcbn.15-143 PMC5018567

[26] Song, Y. *et al.* Insulin sensitivity and insulin secretion determined by homeostasis model assessment and risk of diabetes in a multiethnic cohort of women: The Women’s Health Initiative Observational Study. *Diabetes Care* **30**(7), 1747–1752. 10.2337/dc07-0358 (2007). 17468352 10.2337/dc07-0358 PMC1952235

[27] Gao, L. *et al.* Effect of Perinatal Bisphenol A exposure on serum lipids and lipid enzymes in offspring rats of different sex. *Biomed. Environ. Sci.* **29**(9), 686–689. 10.3967/bes2016.092 (2016). 27806752 10.3967/bes2016.092

[28] Lejonklou, M. H. *et al.* Effects of low-dose developmental Bisphenol A exposure on metabolic parameters and gene expression in male and female Fischer 344 rat offspring. *Environ. Health Perspect.* **125**(6), 067018. 10.1289/EHP505 (2017). 28657538 10.1289/EHP505 PMC5743697

[29] Savage, D. B., Petersen, K. F. & Shulman, G. I. Disordered lipid metabolism and the pathogenesis of insulin resistance. *Physiol. Rev.* **87**(2), 507–520. 10.1152/physrev.00024.2006 (2007). 17429039 10.1152/physrev.00024.2006 PMC2995548

[30] Akash, M. S. H. *et al.* Resveratrol mitigates Bisphenol A-induced metabolic disruptions: Insights from experimental studies. *Molecules (Basel, Switzerland)* **28**(15), 5865. 10.3390/molecules28155865 (2023). 37570835 10.3390/molecules28155865 PMC10421514

[31] Cordiano, R. *et al.* Malondialdehyde as a potential oxidative stress marker for allergy-oriented diseases: An update. *Molecules (Basel, Switzerland)* **28**(16), 5979. 10.3390/molecules28165979 (2023). 37630231 10.3390/molecules28165979 PMC10457993

[32] Dludla, P. V. *et al.* Pancreatic β-cell dysfunction in type 2 diabetes: Implications of inflammation and oxidative stress. *World J. Diabetes* **14**(3), 130–146. 10.4239/wjd.v14.i3.130 (2023). 37035220 10.4239/wjd.v14.i3.130 PMC10075035

[33] Luan, Y. Y. & Yao, Y. M. The clinical significance and potential role of c-reactive protein in chronic inflammatory and neurodegenerative diseases. *Front. Immunol.* **9**, 1302. 10.3389/fimmu.2018.01302 (2018). 29951057 10.3389/fimmu.2018.01302 PMC6008573

[34] Pickles, S., Vigié, P. & Youle, R. J. Mitophagy and quality control mechanisms in mitochondrial maintenance. *Curr. Biol.* **28**(4), R170–R185. 10.1016/j.cub.2018.01.004 (2018). 29462587 10.1016/j.cub.2018.01.004 PMC7255410

[35] Ly, J. D., Grubb, D. R. & Lawen, A. The mitochondrial membrane potential (deltapsi(m)) in apoptosis; an update. *Apoptosis* **8**(2), 115–128. 10.1023/a:1022945107762 (2003). 12766472 10.1023/a:1022945107762

[36] Liu, J. *et al.* Perinatal bisphenol A exposure and adult glucose homeostasis: Identifying critical windows of exposure. *PLoS One* **8**(5), e64143. 10.1371/journal.pone.0064143 (2013). 23675523 10.1371/journal.pone.0064143 PMC3651242

[37] American Diabetes Association. Diagnosis and classification of diabetes mellitus. *Diabetes Care* **30**(1), S42–S47. 10.2337/dc07-S042 (2007). 17192378 10.2337/dc07-S042

[38] Kahn, S. E. The relative contributions of insulin resistance and beta-cell dysfunction to the pathophysiology of Type 2 diabetes. *Diabetologia* **46**(1), 3–19. 10.1007/s00125-002-1009-0 (2003). 12637977 10.1007/s00125-002-1009-0

[39] Dan, T. *et al.* Anti-diabetic effect of Punicagranatum flower polyphenols extract in type 2 diabetic rats: Activation of Akt/GSK-3β and inhibition of IRE1α-XBP1 pathways. *Front. Endocrinol.* **9**, 2018. 10.3389/fendo.2018.00586 (2018). 10.3389/fendo.2018.00586 PMC6196233 30374328

[40] Appleton, D. J., Rand, J. S. & Sunvold, G. D. Plasma leptin concentrations are independently associated with insulin sensitivity in lean and overweight cats. *J. Feline Med. Surg.* **4**(2), 83–93. 10.1053/jfms.2002.0166 (2002). 12027507 10.1053/jfms.2002.0166 PMC10822654

[41] Howard, B. V. Insulin resistance and lipid metabolism. *Am. J. Cardiol.* **84**(1A), 28J-32J. 10.1016/s0002-9149(99)00355-0 (1999). 10418856 10.1016/s0002-9149(99)00355-0

[42] Karadimos, M. J., Kapoor, A., ElKhattabi, I. & Sharma, A. β-cell preservation and regeneration for diabetes treatment: Where are we now?. *Diabetes Manage.* **2**(3), 213–222. 10.2217/dmt.12.21 (2012). 10.2217/dmt.12.21 PMC3462022 23049620

[43] Lei, X. G. & Vatamaniuk, M. Z. Two tales of antioxidant enzymes on β cells and diabetes. *Antioxid. Redox Signal.* **14**(3), 489–503. 10.1089/ars.2010.3416 (2011). 20618069 10.1089/ars.2010.3416 PMC3026656

[44] Gerber, P. A. & Rutter, G. A. The role of oxidative stress and hypoxia in pancreatic beta-cell dysfunction in diabetes mellitus. *Antioxid. Redox Signal.* **26**(10), 501–518. 10.1089/ars.2016.6755 (2017). 27225690 10.1089/ars.2016.6755 PMC5372767

[45] Li, J., Huang, M. & Shen, X. The association of oxidative stress and pro-inflammatory cytokines in diabetic patients with hyperglycemic crisis. *J. Diabetes Complic.* **28**(5), 662–666. 10.1016/j.jdiacomp.2014.06.008 (2014). 10.1016/j.jdiacomp.2014.06.008 25044235

[46] Tabassum, R., Mia, A. R., Reza-Ul-Haq, K. M., Yesmin, M. & Faruqui, J. M. C-reactive protein level in type-2 diabetic patients attending Mymensingh Medical College Hospital. *Mymensingh. Mymensingh Med. J.* **26**(1), 56–60 (2017). 28260756

[47] Cassarino, D. S., Parks, J. K., Parker, W. D. Jr. & Bennett, J. P. Jr. The parkinsonian neurotoxin MPP+ opens the mitochondrial permeability transition pore and releases cytochrome c in isolated mitochondria via an oxidative mechanism. *Biochim. Biophys. Acta* **1453**(1), 49–62. 10.1016/s0925-4439(98)00083-0 (1999). 9989245 10.1016/s0925-4439(98)00083-0

[48] Cnop, M. *et al.* Mechanisms of pancreatic beta-cell death in type 1 and type 2 diabetes: Many differences, few similarities. *Diabetes* **54**(Suppl 2), S97–S107. 10.2337/diabetes.54.suppl_2.s97 (2005). 16306347 10.2337/diabetes.54.suppl_2.s97

[49] Johnson, J. D., Yang, Y. C. & Luciani, D. S. In *) Mechanisms of Pancreatic β-Cell Apoptosis in Diabetes and Its Therapies* (ed. Islam, M.) (Springer, 2014). 10.1007/978-94-007-6884-0_14-2.

[50] Saranya, S., Nair, A. V., Prathapan, P., Neethu, A. S. & Kumar, N. S. Phytochemical analysis of *Centellaasiatica* L. leaf extracts. *Int. J. Adv. Res.* **5**(6), 1828–1832 (2017).

[51] Monton, C., Luprasong, C., Suksaeree, J. & Songsak, T. Validated high performance liquid chromatography for simultaneous determination of stability of madecassoside and asiaticoside in film forming polymeric dispersions. *Rev. Bras. Farmacogn.* **28**, 289–293 (2018).

[52] Mukherjee, S., Das, D., Mukherjee, M., Das, A. S. & Mitra, C. Synergistic effect of folic acid and vitamin B12 in ameliorating arsenic-induced oxidative damage in pancreatic tissue of rat. *J. Nutr. Biochem.* **17**(5), 319–327. 10.1016/j.jnutbio.2005.08.003 (2006). 16214333 10.1016/j.jnutbio.2005.08.003

[53] OECD (2001) OECD Guideline for Testing of Chemicals: Acute Oral Toxicity-Fixed Dose Procedure 420. http://www.oecd.org/document/22/0,2340 (2023).

[54] Alonso-Magdalena, P., Ropero, A. B., Soriano, S., Quesada, I. & Nadal, A. Bisphenol-A: a new diabetogenic factor?. *Hormones (Athens, Greece)* **9**(2), 118–126. 10.1007/BF03401277 (2010). 20687395 10.1007/BF03401277

[55] Giribabu, N., Srinivasarao, N., SwapnaRekha, S., Muniandy, S. & Salleh, N. *Centella asiatica* attenuates diabetes induced hippocampal changes in experimental diabetic rats. *Evid. Based Complement. Alternat. Med.* **2014**, 592062. 10.1155/2014/592062 (2014). 25161691 10.1155/2014/592062 PMC4139016

[56] Neuman, J. C., Truchan, N. A., Joseph, J. W. & Kimple, M. E. A method for mouse pancreatic islet isolation and intracellular cAMP determination. *J. Vis. Exp.* **88**, e50374. 10.3791/50374 (2014). 10.3791/50374 PMC4205887 24998772

[57] Lowry, O. H., Rosebrough, N. J., Farr, A. L. & Randall, R. J. Protein measurement with the Folin phenol reagent. *J. Biol. Chem.* **193**(1), 265–275 (1951). 14907713

[58] Wills, E. D. Evaluation of lipid peroxidation in lipids and biological membranes. In *Biochemical Toxicology: A Practical Approach* (eds Snell, K. & Mullock, B.) 138–140 (IRL Press, 1987).

[59] Sun, Y., Oberley, L. W. & Li, Y. A simple method for clinical assay of superoxide dismutase. *Clin. Chem.* **34**(3), 497–500 (1988). 3349599

[60] Aebi, H. Catalase in vitro. *Methods Enzymol.* **105**, 121–126. 10.1016/s0076-6879(84)05016-3 (1984). 6727660 10.1016/s0076-6879(84)05016-3

[61] Ellman, G. L. Tissue sulfhydryl groups. *Arch. Biochem. Biophys.* **82**, 70–77. 10.1016/0003-9861(59)90090-6 (1959). 13650640 10.1016/0003-9861(59)90090-6

[62] Paglia, D. E. & Valentine, W. N. Studies on the quantitative and qualitative characterization of erythrocyte glutathione peroxidase. *J. Lab. Clin. Med.* **70**(1), 158–169 (1967). 6066618

[63] Cao, C. *et al.* Mild hypothermia ameliorates muscle wasting in septic rats associated with hypothalamic AMPK-induced autophagy and neuropeptides. *Biochem. Biophys. Res. Commun.* **490**(3), 882–888. 10.1016/j.bbrc.2017.06.135 (2017). 28647359 10.1016/j.bbrc.2017.06.135

[64] Bhattacharjee, A. *et al.* Targeting mitochondria with folic acid and vitamin B12 ameliorates nicotine mediated islet cell dysfunction. *Environ. Toxicol.* **33**(9), 988–1000. 10.1002/tox.22586 (2018). 29972271 10.1002/tox.22586

