# Correction: *Centella asiatica* mitigates the detrimental effects of Bisphenol-A (BPA) on pancreatic islets

**DOI:** 10.1038/s41598-025-04486-3

**Published:** 2025-06-13

**Authors:** Oly Banerjee, Siddhartha Singh, Tiyesh Paul, Bithin Kumar Maji, Sandip Mukherjee

**Affiliations:** 1 Department of Physiology, Serampore College, 9 William Carey Road, Serampore, Hooghly, West Bengal 712201 India; 2 Department of Medical Laboratory Technology, School of Allied Health Sciences, Swami Vivekananda University, Bara Kanthalia, West Bengal 700121 India

Correction to: *Scientific Reports* 10.1038/s41598-024-58545-2, published online 05 April 2024

The original version of this Article contained an error in Figure 7a, where the representative flow cytometric data was incorrectly inserted due to inadvertent handling of the data files, and Figure 7b, where representative immunohistochemical data of Bcl2 for BPA100+CA200 group were incorrectly inserted due to inadvertent handling of the data files. The original Figure 7 and accompanying legend appear below.

**Fig. 7:**
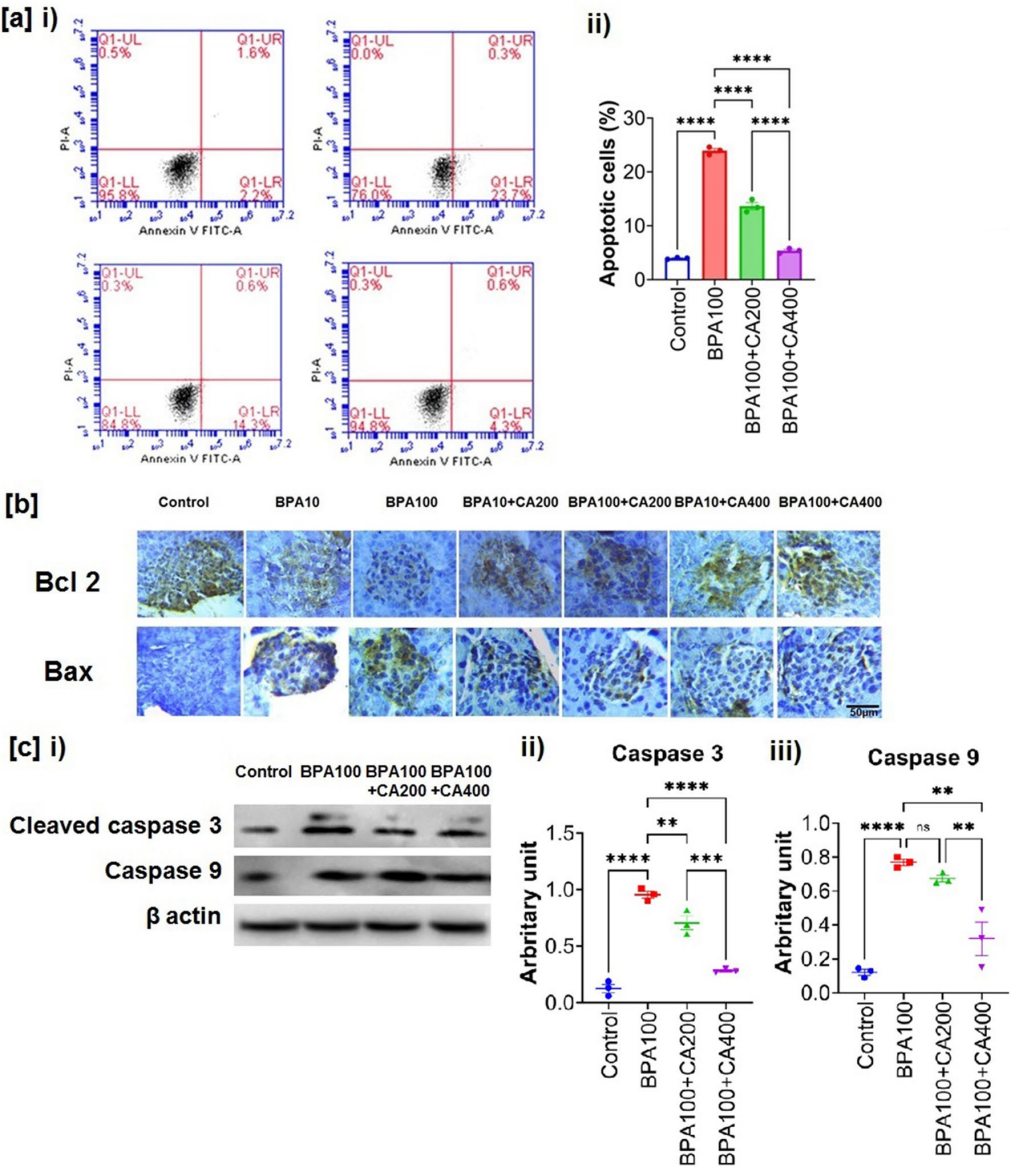
Protective role of CA against BPA-induced apoptosis of pancreatic islet cell and altered expression of Bcl2, Bax, cleaved caspase-3 and caspase-9. Effect of BPA (100 µg/kg body weight for 21 days) on apoptosis of pancreatic islet cells and expression of pro-apoptotic and anti-apoptotic markers with or without supplementation of ethanol extract of CA (200 and 400 mg/kg body weight/day for 21 days). (**a**) i) flow cytometry analysis of apoptosis determined using Annexin V/PI, ii) Apoptotic cells (mean ± SEM, n = 3, for each sample isolated islets were pooled from two animals) of traces shown in (**a**) i). (**b**) i) Representative photomicrograph of immunohischemical analysis of Bcl2 and Bax in pancreatic islets. Results are representative of six mice. Magnification ×200 and scale bar: 50 µm for all panels. (**c**) i) Western blot of cleaved caspase-3 and caspase 9, ii) and iii) Quantification of cleaved caspase-3 (17 kDa) and caspase 9 (mean ± SEM, n = 3, for each sample isolated islets were pooled from two animals). Normality of data was tested by Shapiro–Wilk test. Significance level based on one-way ANOVA, p < 0.05. Significance level based on Tukey’s post hoc test *p < 0.05, **p < 0.01, ***p < 0.001, ****p < 0.0001, *ns* not significant.

The original Article has been corrected.

