# Supplementary material for: Centella asiatica mitigates the detrimental effects of Bisphenol-A (BPA) on pancreatic islets
Source: Sci Rep. 2024 Apr 5;14:8043. doi: 10.1038/s41598-024-58545-2 (PMC10997607; doi:10.1038/s41598-024-58545-2)
Supplement: Supplementary file 1 — Supplementary Information. [file 41598_2024_58545_MOESM1_ESM.docx]

***Centella asiatica* mitigates the detrimental effects of Bisphenol-A (BPA) on pancreatic islets**

Oly Banerjee^a,b^, Siddhartha Singh^a^, Tiyesh Paul^a^, Bithin Kumar Maji^a^, Sandip Mukherjee^a,*^

^a^Department of Physiology, Serampore College, 9 William Carey Road, West Bengal-712201, India.

^b^Department of Medical Laboratory Technology, School of Allied Health Sciences, Swami Vivekananda University, Bara Kanthalia, West Bengal-700121, India

* Corresponding author:

Dr. Sandip Mukherjee

ORCID ID:[0000-0003-4176-3496](javascript:popup_orcidDetail('https://orcid.org','0000-0003-4176-3496');)

Department of Physiology, Serampore College, 9 William Carey Road, Serampore, Hooghly-712201, West Bengal, India. E-mail address: sm_kdc@yahoo.co.in (S. Mukherjee).


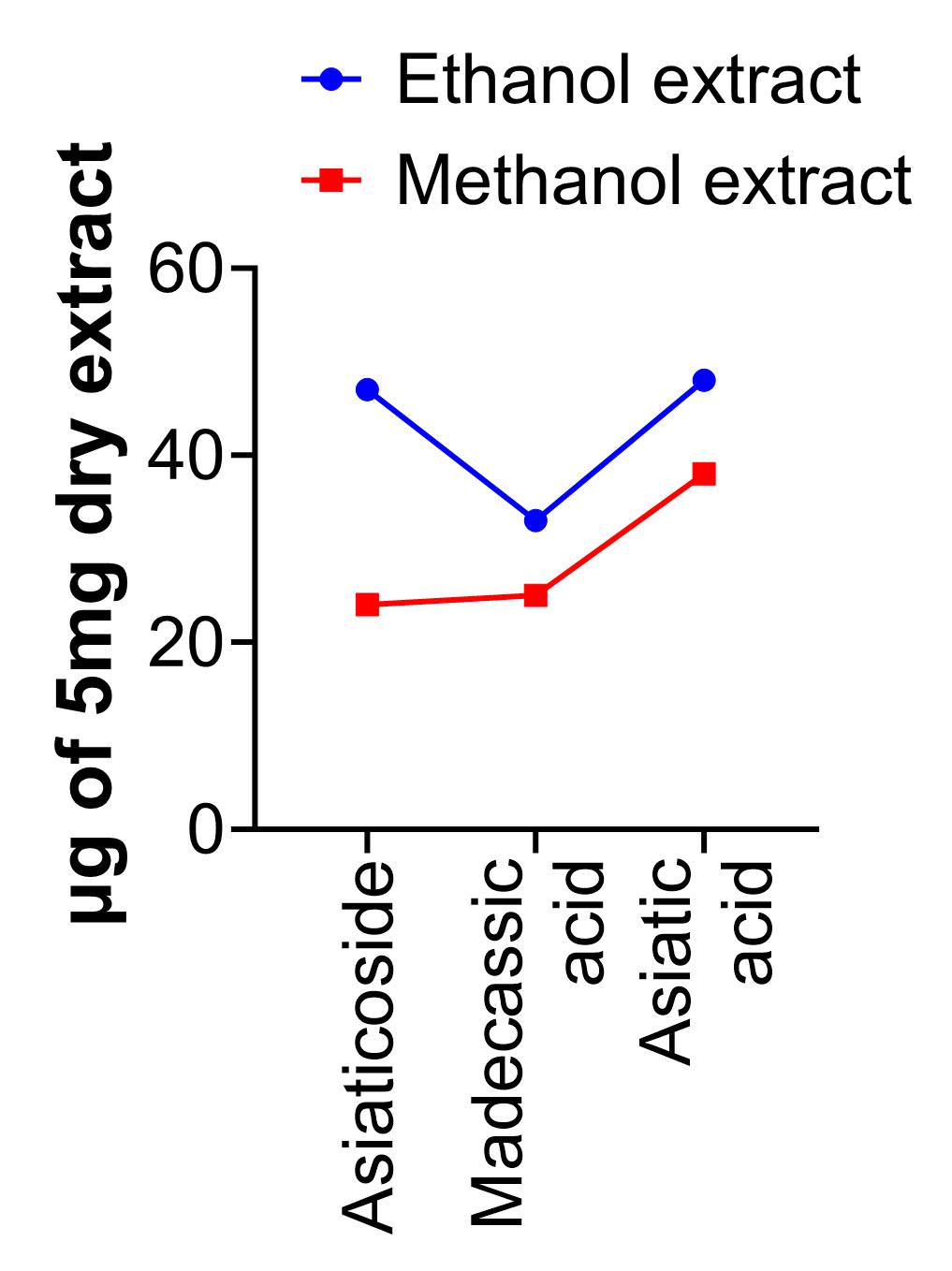


**Figure S1:** Triterpene contents (asiatic acid, asiaticoside and madecassic acid) in ethanol and ethanol extract of *Centella asiatica* as measured by HPLC. Contents of the tritrpenes were presented as μg/5mg of dry extract of *Centella asiatica*.

**Table S1.** **List of different parameters and general signs assessed during toxicity study (n=3)**

| Parameters | Dose of *Centella asiatica* (CA) (mg/kg) | | | | | | |
| --- | --- | --- | --- | --- | --- | --- | --- |
|  | 125 | 250 | 500 | 1000 | 2000 | 3000 | 4000 |
| Body weight | Normal | Normal | Normal | Normal | Normal | Normal | Normal |
| Feed Intake | Normal | Normal | Normal | Normal | Normal | Normal | Normal |
| Eye Colour | No effect | No effect | No effect | No effect | No effect | No effect | No effect |
| Nails Colour | No effect | No effect | No effect | No effect | No effect | No effect | No effect |
| Fur Condition | Normal | Normal | Normal | Normal | Normal | Normal | Normal |
| Aggression | Not present | Not present | Not present | Not present | Not present | Not present | Not present |
| General behavior | Normal | Normal | Normal | Normal | Normal | Normal | Normal |
| Convulsion | Not present | Not present | Not present | Not present | Not present | Not present | Not present |
| Locomotion | No effect | No effect | No effect | No effect | No effect | No effect | No effect |
| Dysponea | Not present | Not present | Not present | Not present | Not present | Not present | Not present |
| Sedation | No effect | No effect | No effect | No effect | No effect | No effect | No effect |

**
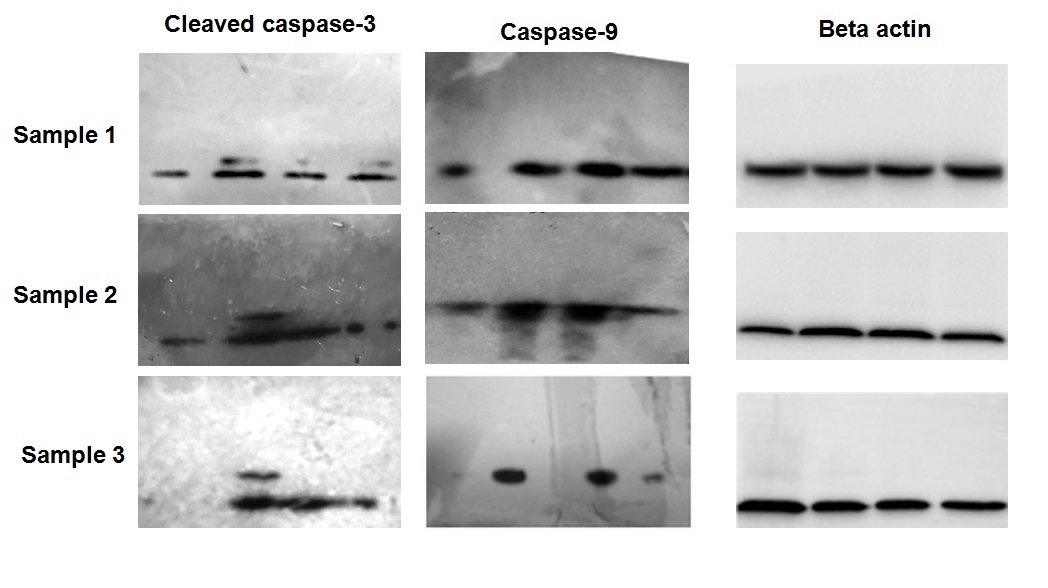
**

**Figure S2: BPA-induced changes in the expression of [a] cleaved caspase 3 and [b] caspase 9 in the pancreatic islets of mice: protective role of CA. Western blot images used for quantitative evaluation of expression of cleaved caspase 3 and caspase 9 in the pancreatic islets of mice as represented in Figure 7cii and 7ciii, respectively.**
